# Genome-wide identification and expression profiling of glutathione S-transferase family under multiple abiotic and biotic stresses in *Medicago truncatula* L.

**DOI:** 10.1371/journal.pone.0247170

**Published:** 2021-02-19

**Authors:** Md. Soyib Hasan, Vishal Singh, Shiful Islam, Md. Sifatul Islam, Raju Ahsan, Amita Kaundal, Tahmina Islam, Ajit Ghosh

**Affiliations:** 1 Department of Biochemistry and Molecular Biology, Shahjalal University of Science and Technology, Sylhet, Bangladesh; 2 Department of Plants, Soils, and Climate, College of Agriculture and Applied Sciences, Utah State University, Logan, Utah, United States of America; 3 Department of Botany, University of Dhaka, Dhaka, Bangladesh; National Botanical Research Institute CSIR, INDIA

## Abstract

Glutathione transferases (GSTs) constitute an ancient, ubiquitous, multi-functional antioxidant enzyme superfamily that has great importance on cellular detoxification against abiotic and biotic stresses as well as plant development and growth. The present study aimed to a comprehensive genome-wide identification and functional characterization of GST family in one of the economically important legume plants—*Medicago truncatula*. Here, we have identified a total of ninety-two putative *MtGST* genes that code for 120 proteins. All these members were classified into twelve classes based on their phylogenetic relationship and the presence of structural conserved domain/motif. Among them, 7 *MtGST* gene pairs were identified to have segmental duplication. Expression profiling of *MtGST* transcripts revealed their high level of organ/tissue-specific expression in most of the developmental stages and anatomical tissues. The transcripts of *MtGSTU*5, *MtGSTU*8, *MtGSTU*17, *MtGSTU*46, and *MtGSTU*47 showed significant up-regulation in response to various abiotic and biotic stresses. Moreover, transcripts of *MtGSTU*8, *MtGSTU*14, *MtGSTU*28, *MtGSTU*30, *MtGSTU*34, *MtGSTU*46 and *MtGSTF*8 were found to be highly upregulated in response to drought treatment for 24h and 48h. Among the highly stress-responsive MtGST members, MtGSTU17 showed strong affinity towards its conventional substrates reduced glutathione (GSH) and 1‐chloro‐2,4‐dinitrobenzene (CDNB) with the lowest binding energy of—5.7 kcal/mol and -6.5 kcal/mol, respectively. Furthermore, the substrate-binding site residues of MtGSTU17 were found to be highly conserved. These findings will facilitate the further functional and evolutionary characterization of *GST* genes in *Medicago*.

## Introduction

Legumes are a pivotal component for ensuring food security and sustainable agriculture worldwide. They provide cereal crops as a source of dietary protein, micronutrients and macronutrients [1–3] as well as many other health-promoting secondary metabolites [4, 5]. But the productivity of legume crops has been significantly decreased due to the exposure of different biotic as well as abiotic stresses in different growing seasons. *Medicago truncatula* was proposed as the first model plants [6] to study rhizobia-legume symbiosis and currently recognized as a model plant for all the legume studies. *M*. *truncatula* is a small diploid (2n = 16) annual legume that has been cultivated widely as a forage plant in the USA for animal feed [7]. Due to its relatively small genome (~375 Mbp) and short generation time (4 months) [7] research has been focused on the critical genes that respond to abiotic and biotic stresses in the *Medicago* [8]. Stresses are often accompanied by an increase in the production of highly toxic reactive oxygen species (ROS) [9]. Overproduction of ROS cause cellular damage, reduce cellular scavenging capacity, unbalance the cellular redox homeostasis; collectively known as oxidative stress [10, 11]. The equilibrium between the production and detoxification of ROS is sustained by the action of various enzymatic and non-enzymatic antioxidants [12, 13]. Glutathione S-transferases (GSTs) are one of the major antioxidant enzymes that play a critical role against abiotic and biotic stresses.

Glutathione S-transferases (GSTs) comprise a large and multifunctional family of enzymes that have importance for their role in phase II detoxification reactions. GST is catalyzing S-conjugation reaction between the thiol group of tri-peptide glutathione, GSH (Glu-Cys-Gly) and electrophilic moiety in the hydrophobic and toxic substrates [14]. They are also involved in the key metabolic steps of many eukaryotic organisms including bacteria and fungi [15, 16]. Apart from these, GSTs play a significant role in the developmental and physiological functions namely hormone biosynthesis, tyrosine degradation, and peroxide breakdown [17], stress signalling [18], nodule function [19], and non-catalytically acting as flavonoid-binding proteins [20]. Recent studies have suggested the involvement of GSTs in the different processes of cell signalling kinases, formation and modulation of ion channels, oxidation-reduction reactions, and post-translational glutathionylation of proteins [21].

GST proteins were classified into 14 distinct classes illustrated as tau, phi, theta, zeta, lambda, γ-subunit of the eukaryotic translation elongation factor 1B (EF1Bγ), dehydroascorbate reductase (DHAR), metaxin, tetrachlorohydroquinone dehalogenase (TCHQD), Ure2p, microsomal prostaglandin E synthase type 2 (mPGES2), hemerythrin, iota, and glutathionyl-hydroquinone reductases (GHR) [22]. Among these 14 classes, five of them including tau, phi, zeta, theta, and TCHQD classes contain a catalytic serine residue and other seven classes such as DHAR, lambda, GHR, mPGES-2, metaxin, iota and hemerythrin contain a very conserved cysteine residue in their active site motif [23]. However, the catalytic nature of EF1Bγ and Ure2p classes remain unclear yet. Based on their cellular location, GSTs could be divided as cytosolic, mitochondrial and microsomal [24]. In plants, most of the cytosolic GSTs typically function as dimer (either homo or hetero form with both subunits originating from the same GST subclass) ranging a molecular weight of 23 to 29 kDa. Each monomer contains a N-terminal thioredoxin-like α/β-domain that binds to GSH moiety (G-site), and a C- terminal α-helical domain, which binds to electrophile substrates (H-site) [25]. These two domains are connected by a short linker sequence of ~10 residues. All the active sites are spreaded over these domains where G site is usually conserved with a highly variable H site to allow a range of hydrophobic substrates [26].

Among the classes of GSTs, phi, tau, theta, and zeta are highly plant-specific while phi and tau are the most abundant form [27]. Overexpression of different member of tau and phi GSTs provided tolerance not only to a range of abiotic stresses, including cold, dehydration, UV, oxidative stress, salt, and heavy metals [28] but also against herbicides [29]. A few members of tau, phi and theta class GST has also possessed glutathione peroxidase activity [25]. Theta class of GSTs play a prominent role to detoxify oxidized lipids [30] whereas zeta GSTs are involved in the tyrosine catabolism [30], tolerance against chilling and salt stresses in *Euphorbia esula* [31]. The DHAR class GSTs are particularly up-regulated during light and drought stresses [32] in comparison to the GHR and mPGES2 members which are differentially regulated under various abiotic stresses in *Arabidopsis* [23].

The functional characterization of *GST* gene family had been performed in various plant species but limited to legumes family. Preliminary genome-wide identification GST members had been performed in *Medicago* [33] with limited genomic information and expression profiling data. In the present study, systematic identification and characterization of *GST* gene family have been conducted in *Medicago* for the understanding of their role in different physiological conditions. The study identified a total of ninety-two *GST* members which add 29 new members and 4 new class of GST as compared with the previous report [33]. Each of these members was analyzed further to identify their physiochemical properties, chromosomal location, presence of conserved motifs, structural organization, sub-cellular localization, phylogenetic relationship, and protein structure. Further, transcript profiling of all *Medicago* GST members was analyzed in different developmental stages, anatomical tissues and response to various abiotic and biotic stress conditions using microarray data and few selected of them were confirmed by quantitative RT-PCR. Additionally, molecular docking study of ten highly stress-responsive members was performed with two well-known substrates of GST- GSH and 1‐chloro‐2,4‐dinitrobenzene (CDNB) to elucidate the binding affinity and residues. This genome-wide analysis and expression profiling will provide a critical platform for the identification of stress and pathogen-resistant genes and development of resistant plants.

## Materials and methods

### Identification, annotation and sequence analysis *GST* genes in *Medicago*

To identify the entire GST family of *Medicago truncatula*, we have performed the systemic BLASTp search of known GST protein sequences of *Arabidopsis* as the query in JGI Phytozome 12 the plant genomic resources *Medicago truncatula* Mt4.0v1 (https://phytozome.jgi.doe.gov/pz/portal.html) with default parameters. All the found hits were further analyzed through the NCBI conserved domain database (https://www.ncbi.nlm.nih.gov/Structure/cdd/wrpsb.cgi) to confirm the presence of conserved GST domains and to classify them in different classes. All the significant hits were analyzed for their annotation, chromosomal location, CDS coordinate (5’ to 3’), length of the respective gene, CDS and protein from Phytozome 12 plant genomic resources. The ExPasy site (http://web.expasy.org/protparam/) was used to calculate their respective molecular weight and isoelectric point (pI) [34]. The subcellular localization of each protein was predicted using CELLO v.2.5: sub-cellular localization predictor (http://cello.life.nctu.edu.tw/) [35], the pSORT prediction software (http://www.genscript.com/wolf-psort.html) [36] and for chloroplast localization ChloroP (http://www.cbs.dtu.dk/services/ChloroP/) [37].

### Chromosomal distribution, gene duplication and Ka/Ks calculation

The chromosomal distribution diagram was plotted using CIRCOS software (http://circos.ca/) [38] according to the chromosome number and CDS coordination information from the Phytozome 12 the plant genomic resources (https://phytozome.jgi.doe.gov/pz/portal). For the duplication study within the *Medicago* genome, data including the synonymous rate (Ks) and non-synonymous rate (Ka) values were retrieved from the plant genome duplication database (http://chibba.agtec.uga.edu/duplication/index/downloads) [39]. Homologous genes were further analyzed by BLASTp search whereas sequence similarities more than 90% among the proteins were considered as segmental duplication [40], and tandem duplicated genes were identified by five or fewer genes in a 100kb region. The selection pressure of duplicated genes was calculated using the Ka/Ks ratio where Ka/Ks ratio >1, = 1, or <1 imply the positive, neutral, and negative selection, respectively. The estimated date (Mya, million years ago) of each duplication event was calculated by using T = Ks/2λ where T is divergence time, Ks is the number of synonymous substitutions per site, and λ is the fixed rate of 1.5×10^−8^ synonymous substitutions per site per year expected for dicotyledonous plants [41].

### Exon-intron structure and molecular evolutions analysis

Gene structure (Exon-intron) map was generated using the gene structure display server, GSDS 2.0 (http://gsds.cbi.pku.edu.cn/) [42]. The individual map was generated by aligning the CDS sequence with their respective genomic DNA sequence. To analyze the evolutionary relationships, protein sequences were aligned using ClustalW to construct an unrooted phylogenetic tree using the default parameters of the maximum likelihood method of MEGA7 software with 1000 bootstraps.

### Identification of conserved motif, SSR markers and glycosylation sites

The online MEME tool (http://meme-suite.org/tools/meme) [43] was used to identify all the conserved motifs among all MtGSTs using default parameters with a maximum number of different motifs to find = 10, minimum width = 10, maximum width = 50. To identify the SSR marker in *MtGSTs*, microsatellite identification tool (MISA, http://pgrc.ipk-gatersleben.de/misa/misa.html) [44] was used with at least ten units for mononucleotide repeats and a minimum five units for dinucleotide, trinucleotide, tetranucleotide, pentanucleotide, and hexanucleotide repeats. The maximum distance between two markers was set 100 units. The number of glycosylation sites in MtGST proteins was identified using NetNGlyc 1.0 server (http://www.cbs.dtu.dk/services/NetNGlyc/) [45].

### Expression profiling of *MtGST* using microarray data

To explore the different temporal and spatial gene expression patterns of the *MtGST* gene family, transcript abundance data of 68 genes with the specific probe was retrieved from genevestigator (https://genevestigator.com/gv/) [46] at various anatomical tissues, developmental stages and in response to different biotic and abiotic stress conditions. Data for seven distinct developmental stages (germination, seedlings, main roots, axillary shoot, developed flower, flower and pods and mature pods) and 24 specific anatomical tissues from primary cells, seedlings, inflorescence, shoot and roots were obtained. Expression data against two devasting abiotic stress (salinity and drought) and in response to six pathogen infection, were also obtained from the same database and analyzed. For salinity stress, 3 days old Jemalong A17 seedlings were treated with 180mM NaCl and radicle samples were collected after 6h, 24h and 48h (Experiment ID: MT-00011). Similarly, 24 days old Jemalong A17 seedlings were exposed to water withholding conditions, and samples were collected after 2d, 3d, 4d, 7d, 11d, 14d and 14d+1d rewatering (Experiment ID: MT-00013). In the case of perturbation, fold change in expression as compared to the respective untreated/control sample was retrieved for each stress conditions. Heat maps with hierarchical clustering were generated by default parameters of Multiple Experiment Viewer (MEV) 4.9 software package with Manhattan correlation [47].

### Analysis of putative promoter of *MtGST* genes

The putative promoter sequences (1kb upstream of the translation start site) of all *MtGSTs* genes were downloaded from the Phytozome version 12.1 database and analyze through PlantCARE (http://bioinformatics.psb.ugent.be/webtools/plantcare/html/) [48] to identify the presence and position of important cis-regulatory elements.

### Plant material and stress treatments

*M*. *truncatula* line Jemalong A-17 (PI670016) seeds obtained from the USDA-GRIN database were used for the analysis. Plants were grown in quart gallon pots filled with a mix of peat moss and perlite (1.5:1) in a growth chamber under controlled conditions with 16 hours of light and 8 hours of dark period. Plants were irrigated with Peter’s solution on alternate days to create optimum growing conditions. The experiment designed for control, drought, and salinity stress with nine replicates each treatment. For salinity treatment, three weeks old plants irrigated with 180mM NaCl in Peter’s solution. To avoid osmotic shock/stress, the salinity level was increased in two steps. A solution of 300mM Mannitol and 20% Poly-Ethylene Glycol (PEG6000) in Peter’s solution was used to create drought conditions. The control plants were irrigated with Peter’s solution. The leaf and root samples were collected after 24 and 48 hours of the treatment from three individual plants in each treatment group for RNA extraction and gene expression analysis.

### Quantitative reverse transcription-PCR (qRT-PCR)

The expression analyses of selected 10 *MtGST* genes (S1 Table in S2 File) were performed using qRT-PCR. RNA extraction was done with TRIzol reagent (Invitrogen, Carlsbad, CA, USA). DNA contamination was removed by treating RNA with TURBO DNase enzyme (Invitrogen, Carlsbad, CA, USA). The gene expression was done by qRT-PCR in the BIO-RAD CFX Connect Real-Time System (BIO-RAD, Hercules, CA, USA). The iTaq Universal SYBR Green One-Step Kit (BIO-RAD, Hercules, CA, USA) was used in the study. The reaction was carried out in 10μl reaction volume containing 25 ng of RNA, 0.25 μM of each primer, 0.125 μl iScript Reverse Transcriptase enzyme (BIO-RAD, Hercules, CA, USA), and 5 μl of 2x one-step SYBR Green Reaction mix (BIO-RAD, Hercules, CA, USA). The PCR program was as follows: 50°C for 10 min, 95°C for 1 min, then 40 cycles of 95°C denaturation for 10 s, 60°C annealing and extension for 20 s. The *Medicago PDF2*, *PPRrep*, and *Ubiquitin* genes were used as reference for the study (S1 Table in S2 File). The amplification specificity was tested with melt curve analysis as follows: 65–95°C, 0.5°C increments 2–5 second per step. Two technical replicates were used for each biological replicate, so a total of six replicates for each treatment were used for analysis. The gene expression data analysis was done using CFX manager software [49].

### Homology based modelling of few selected MtGST proteins

Three-dimensional structure of 10 highly stress up-regulated members were created using the automated homology-based modelling tool of SWISS-MODEL (http://swissmodel.expasy.org/) [51]. The structure of MtGSTU8 and MtGSTU17 were built using the template of *Glycine max* GSTU (PDB: 4TOP), MtGSTU28 and MtGSTU29 using a synthetic GST tau protein (PDB: 6GHF), MtGSTU46 and MtGSTU47 using a glutathione transferase family member from *Ricinus communis* (PDB: 4J2F), MtGSTF8 using *Arabidopsis thaliana* GSTF7 (PDB: 6EZY), MtGSZ2 and MtGSTZ3 using a Zeta class glutathione S-transferase from *Arabidopsis thaliana* (PDB: 1E6B), and finally structure of MtGSTT2 was built based on the human GSTT1-1 (PDB: 2C3N). All these structures and putative active site residues were visualized using BIOVIA Discovery Studio Visualizer v.4.5.

### Molecular Docking study

The 3D structure of the above MtGST proteins was used as a receptor to check the binding potential with two well-characterized substrates- reduced glutathione (GSH) and 1-Chloro-2,4-dinitrobenzene (CDNB). Three-dimensional chemical structures of these two ligands were retrieved from the PUBCHEM compound database (http://www.pubchem.ncbi.nlm.nih.gov) as SDF file. The ligand file conversions required for the docking study were performed using the open-source chemical toolbox Open Babel v. 2.3.2. [52]. Grid box parameters were set to accommodate each compound within the binding site of each protein and determined using AutoDock Tools v. 1.5.6rc3 [53]. Molecular docking calculations for two ligands with each of the proteins were performed using AutoDock Vina v. 1.1.2. [54] and the PDBQT files were generated using the MGL tools.

### Statistical analysis

Statistical data analysis was performed for the relative normalized expression from three biological replicates under each treatment and time-point (n = 6). The Student’s t-test were performed for each treatment against the respective controls to determine the significant alteration (P value< 0.05) that were marked with different letters.

## Results

### *In silico* identification, nomenclature, and characterization of *M*. *truncatula GST* transcripts

After a systematic BLASTp search against the *Medicago truncatula* Mt4.0v1 genome database with the query sequence of *Arabidopsis* GST proteins [55], a total of 120 non-redundant proteins encoded by 92 genes were obtained containing either typical GST N- and/or C-terminal domains. All these full-length putative GST proteins were classified into twelve families based on their conserved domain analysis. The GST members of *Medicago* were named according to the previously reported Dixon and Edwards, 2010 [25] by adding prefix “Mt” (*Medicago truncatula*) to the subclass identifiers (e.g., MtGSTU, MtGSTF, MtGSTT, MtGSTZ, MtGSTL, MtTCHQD, MtDHAR, MtEF1Bγ, MtMGST, MtGHR, MtGSTM, MtGSTH represents tau, phi, theta, zeta, lambda, TCHQD, DHAR, EF1Bγ, mPGES, GHR, metaxin, hemerythrin class, respectively) followed by gene number. Previously identified MtGST members were assigned the same name as given by Han et al. (2018), and the newly identified members were added after them. Previously identified *MtGSTU*48 is excluded in our study because of the absence of conserved GST domains. The tau and phi classes are found to be the most abundant with 51 and 11 members, respectively. The length of the *MtGST* genes ranged from 210 bp (*MtGSTF*10) to 15125 bp (*MtGSTL*5), and the deduced complementary DNA sequence (CDS) were 210 bp (*MtGSTF*10) to 3816 bp (*MtGSTH3*) long, whereas the polypeptide length varied from 69 aa (MtGSTF10) to 1271 aa (MtGSTH3). The molecular weight (MW) of the MtGST proteins varied from the lowest 7.90 kDa (MtGSTF10) to the highest of 146.59 kDa (MtGSTH3) and the predicted pI values ranged from 4.86 to 9.48. However, the average gene length, CDS, protein, MW and PI were 2892.15 bp, 845.67 bp, 280.94 aa, 32.150 kDa and 6.473, respectively (Table 1). Most of the MtGST proteins were found to be 200 to 400 aa long with few exceptions of longer proteins (MtGSTL5 and MtGSTH3-5) and shorter proteins (MtGSTU50-52 and MtGSTF10) due to the presence of other domains apart from GST and truncated/deleted genes, respectively. Most of the MtGST proteins were predicted to be localized in the cytoplasm, followed by chloroplast, mitochondria, nucleus, plasma membrane and extracellular space (Table 1).

**Table 1:** List of identified GST members in *Medicago trancatula* along with their detailed information and subcellular localization.

Sl no	Gene Name	Locus Name	Protein variants	CDS coordinate (5’ - 3’)	Strand	Length			MW (kDa)	pI	Localization
						Gene	CDS	PP			
1	*MtGSTU*1	Medtr1g090060	Medtr1g090060.1	40324849–40326324	-	1476	678	225	26.43	5.81	Cy^a^^,^^b^
2	*MtGSTU*2	Medtr1g090070	Medtr1g090070.1	40329535–40331502	-	1968	690	229	27.07	6.26	Cy^a^^,^^b^
3	*MtGSTU*3	Medtr1g090090	Medtr1g090090.1	40336885–40338619	-	1735	678	225	26.54	6.26	Cy^a^^,^^b^
4	*MtGSTU*4	Medtr1g090100	Medtr1g090100.1	40342187–40343905	-	1719	666	221	26.02	5.68	Cy^a^^,^^b^
5	*MtGSTU*5	Medtr1g090150	Medtr1g090150.1	40382849–40384473	+	1625	681	226	26.44	6.07	Cy^a^^,^^b^
6	*MtGSTU*6	Medtr1g115195	Medtr1g115195.1	51864615–51865729	+	1115	666	221	25.80	6.39	Cy^a^^,^^b^
7	*MtGSTU*7	Medtr2g070060	Medtr2g070060.1	29488070–29489572	+	1503	660	219	25.57	5.60	Cy^a^, Nu^b^
8	*MtGSTU*8	Medtr2g070070	Medtr2g070070.1	29490218–29492164	+	1947	660	219	25.67	5.76	Cy^a^^,^^b^
9	*MtGSTU*9	Medtr2g070110	Medtr2g070110.1	29502243–29504071	+	1829	657	219	25.79	5.78	Cy^a^, Nu^b^
10	*MtGSTU*10	Medtr2g070120	Medtr2g070120.1	29504938–29505698	-	761	660	219	25.47	6.14	Cy^a^^,^^b^
11	*MtGSTU*11	Medtr2g070130	Medtr2g070130.1	29506719–29507688	-	970	660	219	25.31	5.89	Cy^a^^,^^b^
12	*MtGSTU*12	Medtr2g070140	Medtr2g070140.1	29509921–29511319	-	1399	612	203	23.65	8.25	Cy^a^^,^^b^
13	*MtGSTU*13	Medtr2g070150	Medtr2g070150.1	29516864–29518456	-	1593	612	204	23.78	6.02	Cy^a^^,^^b^
14	*MtGSTU*14	Medtr2g070180	Medtr2g070180.1	29534192–29535647	-	1456	654	217	25.32	6.46	Cy^a^^,^^b^
15	*MtGSTU*15	Medtr2g070200	Medtr2g070200.1	29540260–29541815	-	1556	666	221	25.78	8.24	Cy^a^^,^^b^
16	*MtGSTU*16	Medtr2g070210	Medtr2g070210.1	29544065–29549359	-	5295	666	221	25.69	5.19	Cy^a^^,^^b^
17	*MtGSTU*17	Medtr3g467420	Medtr3g467420.1	27821302–27823017	+	1716	675	224	25.97	6.61	Cy^a^^,^^b^
18	*MtGSTU*18	Medtr3g467430	Medtr3g467430.1	27824358–27826866	+	2509	675	224	26.42	6.61	Cy^a^, Nu^b^
19	*MtGSTU*19	Medtr3g099757	Medtr3g099757.1	45742035–45743058	-	1024	672	223	25.55	5.16	Cy^a^^,^^b^
20	*MtGSTU*20	Medtr4g019780	Medtr4g019780.1	6269250–6271309	+	2060	663	220	25.28	5.20	Cy^a^^,^^b^
21	*MtGSTU*21	Medtr4g019790	Medtr4g019790.1	6273359–6274845	+	1487	678	225	25.75	7.81	Cy^a^^,^^b^
22	*MtGSTU*22	Medtr4g059730	Medtr4g059730.1	22039486–22040644	+	1159	657	218	25.38	5.67	Cy^a^, Cp^b^
23	*MtGSTU*23	Medtr4g124130	Medtr4g124130.1	51265312–51266610	-	1299	663	220	25.71	5.93	Cy^a^^,^^b^
24	*MtGSTU*24	Medtr5g037380	Medtr5g037380.1	16368718–16370307	-	1590	687	228	25.36	5.39	Cy^a^, Cp^b^
25	*MtGSTU*25	Medtr5g040430	Medtr5g040430.1	17781983–17783604	+	1622	678	225	26.08	5.64	Cy^a^^,^^b^
26	*MtGSTU*26	Medtr5g076900	Medtr5g076900.1	32800849–32802657	-	1809	666	221	25.15	5.54	Cy^a^^,^^b^
27	*MtGSTU*27	Medtr6g080440	Medtr6g080440.1	30347740–30350780	-	3041	672	223	25.37	4.86	Cy^a^, Cp^b^
28	*MtGSTU*28	Medtr7g065230	Medtr7g065230.1	23789741–23792149	-	2409	660	219	15.59	5.31	Cy^a^^,^^b^
29	*MtGSTU*29	Medtr7g065260	Medtr7g065260.1	23796391–23798295	-	1905	699	235	26.72	5.22	Cy^a^, Cp^b^
30	*MtGSTU*30	Medtr7g065265	Medtr7g065265.1	23800564–23802857	-	2295	675	224	26.13	5.75	Cy^a^^,^^b^
31	*MtGSTU*31	Medtr7g065270	Medtr7g065270.1	23805240–23808449	-	3210	675	224	26.23	5.15	Cy^a^^,^^b^
32	*MtGSTU*32	Medtr7g065290	Medtr7g065290.1	23814968–23817913	-	2946	675	224	26.15	5.62	Cy^a^, Cp^b^
33	*MtGSTU*33	Medtr7g065590	Medtr7g065590.1	23820654–23823642	-	2989	675	224	26.11	5.32	Cy^a^, Cp^b^
34	*MtGSTU*34	Medtr7g065600	Medtr7g065600.1	23826932–23828482	-	1551	675	224	25.88	5.89	Cy^a^, Cp^b^
35	*MtGSTU*35	Medtr7g065630	Medtr7g065630.1	23837589–23838924	+	1336	720	239	27.53	7.73	Cy^a^, Cp^b^
36	*MtGSTU*36	Medtr7g065640	Medtr7g065640.1	23839534–23840910	-	1377	624	207	23.92	6.00	Cy^a^^,^^b^
37	*MtGSTU*37	Medtr7g065660	Medtr7g065660.1	23843336–23844576	-	1241	678	225	26.11	5.27	Cy^a^, Cp^b^
38	*MtGSTU*38	Medtr7g065680	Medtr7g065680.1	23848890–23851165	+	2276	666	221	25.50	5.59	Cy^a^^,^^b^
39	*MtGSTU*39	Medtr7g065700	Medtr7g065700.1	23852761–23853724	+	964	681	226	25.92	5.66	Cy^a^^,^^b^
40	*MtGSTU*40	Medtr7g065710	Medtr7g065710.1	23855145–23856094	+	950	672	223	25.62	8.74	Cy^a^, Cp^b^
41	*MtGSTU*41	Medtr7g065720	Medtr7g065720.1	23856633–23857592	-	960	678	225	25.97	5.51	Cy^a^^,^^b^
42	*MtGSTU*42	Medtr7g065740	Medtr7g065740.1	23860316–23861627	-	1312	681	226	26.18	5.90	Cy^a^, Cp^b^
43	*MtGSTU*43	Medtr7g065750	Medtr7g065750.1	23864218–23865155	-	938	681	226	26.22	6.01	Cy^a^, Nu^b^
44	*MtGSTU*44	Medtr8g056940	Medtr8g056940.1	18873208–18874964	-	1757	660	219	25.70	5.26	Cy^a^^,^^b^
45	*MtGSTU*45	Medtr8g061950	Medtr8g061950.1	25866267–25867866	-	1600	702	233	26.82	5.50	Cy^a^^,^^b^
46	*MtGSTU*46	Medtr8g087410	Medtr8g087410.1	36120245–36121742	+	1498	678	225	26.20	6.97	Cy^a^, Nu^b^
47	*MtGSTU*47	Medtr8g087425	Medtr8g087425.1	36133606–36135117	+	1512	675	224	25.91	5.68	Cy^a^, Cp^b^
48	*MtGSTU*49	Medtr0186s0030	Medtr0186s0030.1	7735–9506	-	1772	426	141	16.31	5.54	Cy^a^^,^^b^
49	*MtGSTU*50	Medtr1g110250	Medtr1g110250.1	49721325–49721645	-	321	243	80	9.36	8.66	Mt^a^, Cy^b^
50	*MtGSTU*51	Medtr2g072120	Medtr2g072120.1	30255927–30256730	-	804	231	76	9.20	9.48	Mt^a^, Cy^b^
51	*MtGSTU*52	Medtr5g037390	Medtr5g037390.1	16372991–16374241	-	1251	297	98	11.42	8.93	Mt^a^, Cy^b^
52	*MtGSTF*1	Medtr1g026140	Medtr1g026140.1	8446317–8447711	-	1395	669	222	25.20	8.64	Mt^a^, Cy^a^, Cp^b^
53	*MtGSTF*2	Medtr1g088825	Medtr1g088825.1	39755058–39756904	-	1847	645	214	24.37	5.99	Cy^a^, Cp^b^
54	*MtGSTF*3	Medtr1g088840	Medtr1g088840.1	39760559–39762258	-	1700	645	214	24.379	5.99	Cy^a^, Cp^b^
			Medtr1g088840.2	39760702–39762079	-	1378	504	167	18.96	5.61	Cy^a^^,^^b^
			Medtr1g088840.3	39760702–39762079	-	1378	357	118	13.46	5.61	Cy^a^^,^^b^, Mt^a^
55	*MtGSTF*4	Medtr1g088845	Medtr1g088845.1	39770254–39771492	-	1239	645	214	24.28	5.71	Cy^a^, Pm^a^, Cp^b^
56	*MtGSTF*5	Medtr1g088850	Medtr1g088850.1	39774932–39776217	+	1286	663	220	25.17	5.26	Cy^a^, Cp^b^
57	*MtGSTF6*	Medtr3g450790	Medtr3g450790.1	17572206–17574321	-	2116	648	215	24.85	5.91	Cy^a^, Mt^b^
58	*MtGSTF*7	Medtr3g064700	Medtr3g064700.1	29161367–29163606	-	2240	642	213	24.22	6.10	Cy^a^^,^^b^
59	*MtGSTF*8	Medtr5g090910	Medtr5g090910.1	39600426–39601735	+	1310	651	216	24.81	6.18	Cy^a^^,^^b^
			Medtr5g090910.2	39600426–39601735	+	1310	480	159	18.33	6.21	Cy^a^, Nu^b^
60	*MtGSTF*9	Medtr5g090920	Medtr5g090920.1	39610753–39612745	+	1993	651	216	24.91	5.63	Cy^a^^,^^b^
61	*MtGSTF*10	Medtr1g492670	Medtr1g492670.1	41527339–41527548	-	210	210	69	7.9	9.40	Mt^a^, Cy^b^
62	*MtGSTF*11	Medtr3g450930	Medtr3g450930.1	17644290–17647516	+	3227	648	215	25.51	5.74	Cy^a^^,^^b^
63	*MtEF1Bγ*1	Medtr2g005570	Medtr2g005570.1	213617–217636	+	4020	1257	418	47.72	6.43	Cy^a^^,^^b^
			Medtr2g005570.2	213718–217571	+	3854	1257	418	47.72	6.43	Cy^a^^,^^b^
64	*MtEF1Bγ*2	Medtr3g058940	Medtr3g058940.1	23532181–23535612	-	3432	1260	419	47.74	6.00	Cy^a^^,^^b^
65	*MtEF1Bγ*3	Medtr4g134770	Medtr4g134770.1	56487455–56490004	+	2550	1260	419	47.99	6.00	Cy^a^^,^^b^
			Medtr4g134770.2	56487504–56489959	+	2456	1155	384	44.10	5.88	Cy^a^^,^^b^
66	*MtEF1Bγ*4	Medtr1568s0020	Medtr1568s0020.1	538–1167	-	630	630	210	23.41	6.30	Mt^a^, Pm^a^, Cp^b^
67	*MtDHAR*1	Medtr1g115500	Medtr1g115500.1	52187447–52191417	-	3971	639	212	23.30	5.58	Cp^a^, Cy^a^^,^^b^
68	*MtDHAR*2	Medtr3g066060	Medtr3g066060.1	29842736–29848501	+	5766	795	264	29.48	6.24	Cp^a^^,^^b^^,^^c^
69	*MtGSTL*1	Medtr1g067170	Medtr1g067170.1	28913203–28916342	-	3140	735	244	27.82	6.17	Cy^a^^,^^b^
70	*MtGSTL*2	Medtr1g067180	Medtr1g067180.1	28920980–28923650	-	2671	714	237	27.04	5.88	Cy^a^, Cp^b^^,^^c^
			Medtr1g067180.2	28920982–28923571	-	2590	534	177	19.87	5.59	Ec^a^, Cp^b^^,^^c^
			Medtr1g067180.3	28920980–28923571	-	2592	705	234	27.01	5.42	Cp^a^^,^^b^^,^^c^, Cy^a^
			Medtr1g067180.4	28921512–28923571	-	2060	651	216	24.46	5.37	Cp^a^^,^^b^^,^^c^, Cy^a^
			Medtr1g067180.5	28920982–28923571	-	2590	585	194	21.94	6.07	Ec^a^, Cp^b^^,^^c^
71	*MtGSTL*3	Medtr1g116270	Medtr1g116270.1	52557070–52559696	+	2677	933	310	35.62	7.22	Cp^a^^,^^b^^,^^c^
72	*MtGSTL*4	Medtr7g100320	Medtr7g100320.1	40287456–40289780	-	2325	711	236	26.99	5.46	Cy^a^^,^^b^
73	*MtGSTL*5	Medtr1g067150	Medtr1g067150.1	28895825–28910918	-	15094	3501	1166	133.09	5.10	Cy^a^, Nu^a^, Cp^b^
			Medtr1g067150.2	28895794–28910918	-	15125	3501	1166	133.09	5.10	Cy^a^, Nu^a^, Cp^b^
74	*MtGSTT*1	Medtr8g098420	Medtr8g098420.1	40882292–40885154	-	2863	753	250	28.30	9.43	Mt^a^^,^^b^, Cy^a^
			Medtr8g098420.2	40882292–40885154	-	2863	567	188	20.95	8.93	Pm^a^, Cy^b^
75	*MtGSTT*2	Medtr8g098430	Medtr8g098430.1	40879266–40882266	-	3001	759	252	28.89	7.85	Cy^a^^,^^b^, Nu^a^, Mt^a^
76	*MtGSTZ*1	Medtr4g134370	Medtr4g134370.1	56258117–56263708	-	5592	693	230	26.26	6.31	Cy^a^^,^^b^, Mt^a^
			Medtr4g134370.2	56258117–56263708	-	5592	693	230	26.26	6.31	Cy^a^^,^^b^, Mt^a^
			Medtr4g134370.3	56258117–56263708	-	5592	693	230	26.26	6.31	Cy^a^^,^^b^, Mt^a^
77	*MtGSTZ*2	Medtr4g134380	Medtr4g134380.1	56264973–56268969	+	3997	681	226	25.73	6.45	Mt^a^, Cy^a^^,^^b^
			Medtr4g134380.2	56264973–56268969	+	3997	534	177	20.05	7.72	Mt^a^, Pm^a^, Cy^b^
78	*MtGSTZ*3	Medtr4g134360	Medtr4g134360.1	56253533–56257371	+	3839	420	139	16.06	8.55	Mt^a^, Pm^a^, Cy^b^
			Medtr4g134360.2	56253570–56256522	+	2953	420	139	16.06	8.55	Mt^a^, Pm^a^, Cy^b^
			Medtr4g134360.3	56253530–56257386	+	3857	420	139	16.06	8.55	Mt^a^, Pm^a^, Cy^b^
			Medtr4g134360.4	56253533–56257371	+	3839	420	139	16.06	8.55	Mt^a^, Pm^a^, Cy^b^
79	*MtTCHQD*1	Medtr3g088635	Medtr3g088635.1	40356624–40359320	-	2697	804	267	31.61	8.95	Nu^a^, Cy^b^
			Medtr3g088635.2	40356624–40359320	-	2697	711	236	28.09	9.14	Nu^a^, Cy^b^
			Medtr3g088635.3	40356624–40358679	-	2056	711	236	28.09	9.14	Nu^a^, Cy^b^
			Medtr3g088635.4	40356624–40358679	-	2056	804	267	31.61	8.95	Nu^a^, Cy^b^
80	*MtMGST*1	Medtr5g076470	Medtr5g076470.1	32625821–32629476	+	3656	453	150	16.93	9.39	Pm^a^, Cp^b^
			Medtr5g076470.2	32625821–32629476	+	3656	441	146	16.57	9.16	Pm^a^, Cp^b^
81	*MtMGST*2	Medtr3g005720	Medtr3g005720.1	340338–346010	-	5673	972	323	36.14	6.55	Cp^a^^,^^b^^,^^c^, Nu^a^
82	*MtMGST*3	Medtr3g114070	Medtr3g114070.1	53256905–53259598	+	2694	969	322	36.41	9.08	Mt^a^, Cp^a^^,^^b^^,^^c^
83	*MtMGST*4	Medtr6g069420	Medtr6g069420.1	24981419–24987900	+	6482	978	325	36.31	8.76	Cp^a^^,^^b^^,^^c^
84	*MtGHR*1	Medtr1g069575	Medtr1g069575.1	30229576–30231346	-	1771	1224	407	45.51	8.58	Mt^a^, Cp^b^^,^^c^
85	*MtGHR*2	Medtr4g084040	Medtr4g084040.1	32721844–32724728	-	2885	1068	355	41.07	6.56	Nu^a^, Cp^b^
			Medtr4g084040.2	32721836–32724734	-	2899	978	325	37.47	5.78	Mt^a^, Cp^a^, Cy^b^
			Medtr4g084040.3	32721836–32724737	-	2902	978	325	37.47	5.78	Mt^a^, Cp^a^, Cy^b^
			Medtr4g084040.4	32721836–32724728	-	2893	978	325	37.47	5.78	Mt^a^, Cp^a^, Cy^b^
			Medtr4g084040.5	32721836–32724734	-	2899	978	325	37.47	5.78	Mt^a^, Cp^a^, Cy^b^
			Medtr4g084040.6	32721836–32724734	-	2899	780	259	30.12	5.26	Cy^a^^,^^b^, Nu^b^
			Medtr4g084040.7	32721836–32724734	-	2899	780	259	30.12	5.26	Cy^a^^,^^b^, Nu^b^
86	*MtGSTM*1	Medtr2g036910	Medtr2g036910.1	16012913–16019765	+	6853	993	330	36.97	5.08	Nu^a^, Cy^a^^,^^b^
87	*MtGSTM*2	Medtr4g127630	Medtr4g127630.1	53027056–53030193	-	3138	945	314	35.19	5.88	Cy^a^^,^^b^
88	*MtGSTH*1	Medtr1g050385	Medtr1g050385.1	19323435–19326258	+	2824	1047	348	39.48	5.38	Cy^a^, Nu^a^^,^^b^
89	*MtGSTH*2	Medtr1g111960	Medtr1g111960.1	50627457–50630153	+	2697	999	332	37.53	6.24	Cy^a^^,^^b^
90	*MtGSTH*3	Medtr4g107440	Medtr4g107440.1	44453747–44465290	+	11544	3816	1271	146.59	6.35	Nu^a^^,^^b^
91	*MtGSTH*4	Medtr6g083900	Medtr6g083900.1	31304656–31314509	+	9854	3705	1234	138.56	5.60	Nu^a^^,^^b^
92	*MtGSTH*5	Medtr8g104410	Medtr8g104410.1	43980913–43994829	+	13917	3732	1243	140.60	5.84	Nu^a^^,^^b^
			Medtr8g104410.2	43986906–43994829	+	7924	3024	1007	114.00	5.95	Nu^a^^,^^b^

Abbreviations: CDS (bp), coding DNA Sequence; PP (aa), Polypeptide; MW, Molecular Weight; pI, Isoelectric point; bp, base pair; aa, amino acid; kDa, kilodalton; Cp, Chloroplast; Ec, Extracellular; Cy, Cytoplasm; Mt, Mitochondria; Nu, Nucleus; Pm, Plasma-membrane.

^a^Localization prediction by CELLO v.2.5 (http://cello.life.nctu.edu.tw/)

^b^Localization prediction by pSORT (http://www.genscript.com/wolf-psort.html)

^c^Chloroplast localization signal confirmed by ChloroP (http://www.cbs.dtu.dk/services/ChloroP/)

### Chromosomal localization and gene duplication

**Fig 1:**
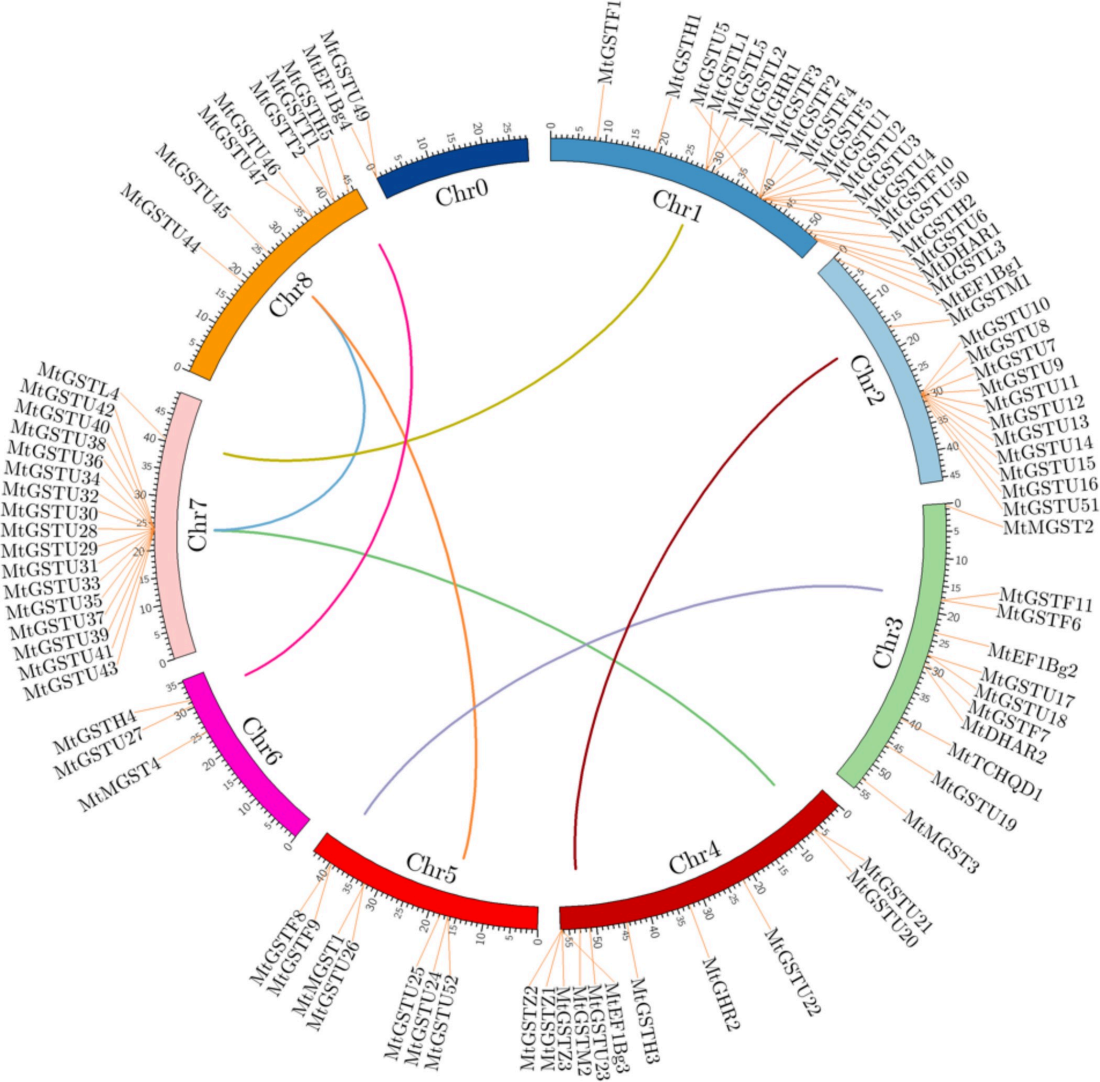
Chromosomal distribution and duplication analysis of *MtGST* genes. In the diagram, eight chromosomes and one scaffold region (Chr 0) are represented in partial circles with different colours. *MtGST* genes in different chromosomes are indicated by red labels. Different coloured lines connecting two chromosomal regions indicate the duplicated gene pairs in *Medicago*. The illustration was generated using CIRCOS software.

The 92 non-redundant *MtGST* genes were mapped on the 8 different chromosomes and scaffold regions of *M*. *truncatula* (Fig 1). The number of *MtGST* genes on each chromosome varied widely. Chromosome 1 contained the highest number of twenty-one *GST* members, followed by chromosome 7 and 2 with seventeen and thirteen members, respectively. In chromosome 3 and 4 has eleven genes each, while chromosome 5 and 8 have seven genes each. The lowest number of three members were located in chromosome 6. To deduce the intensified number of *MtGST* gene family members, the gene duplication events were investigated. A total of seven gene pairs were found to be duplicated- *MtGSTU*21 and *MtGSTU*30, *MtGSTU*40 and *MtGSTU*45, *MtGSTU*24 and *MtGSTU*45, *MtGSTF*6 and *MtGSTF*8, *MtGSTL*4 and *MtGSTL*5, *MtGSTM*1 and *MtGSTM*2, and *MtGSTH*4 and *MtGSTH*5. All of them are possessing segmental duplication type (Table 2). To analyze the selection pressure among the duplicated gene pairs, the ratio of nonsynonymous (Ka) to synonymous (Ks) values were calculated which showed that all of the duplicated genes were negatively selected. All the duplicated *MtGST* gene pairs have a Ka/Ks values less than 1 (Table 2), suggesting that all of these gene pairs evolved through purifying selection. Additionally, the divergence time of duplication was varied from 22.31 to 55.94 Mya (Table 2).

**Table 2:** Duplicated *GST* genes and the probable dates of duplication in *Medicago*.

Gene 1	Gene 2	Ka	Ks	Ka/Ks	Duplication Time (Mya)	Purifying selection	Duplicate Type
*MtGSTU*21	*MtGSTU*30	0.2854	1.6782	0.1700	55.94	Yes	Segmental
*MtGSTU*40	*MtGSTU*45	0.3375	0.8994	0.3752	29.97	Yes	Segmental
*MtGSTU*24	*MtGSTU*45	0.3375	0.8994	0.3752	29.97	Yes	Segmental
*MtGSTF*6	*MtGSTF*8	0.1989	1.3808	0.1440	46.02	Yes	Segmental
*MtGSTL*4	*MtGSTL*5	0.1364	0.5991	0.2276	19.97	Yes	Segmental
*MtGSTM*1	*MtGSTM*2	0.2402	0.6693	0.3588	22.31	Yes	Segmental
*MtGSTH*4	*MtGSTH*5	0.1080	0.5677	0.1902	18.92	Yes	Segmental

### *MtGST* family members are evolutionary conserved

**Fig 2:**
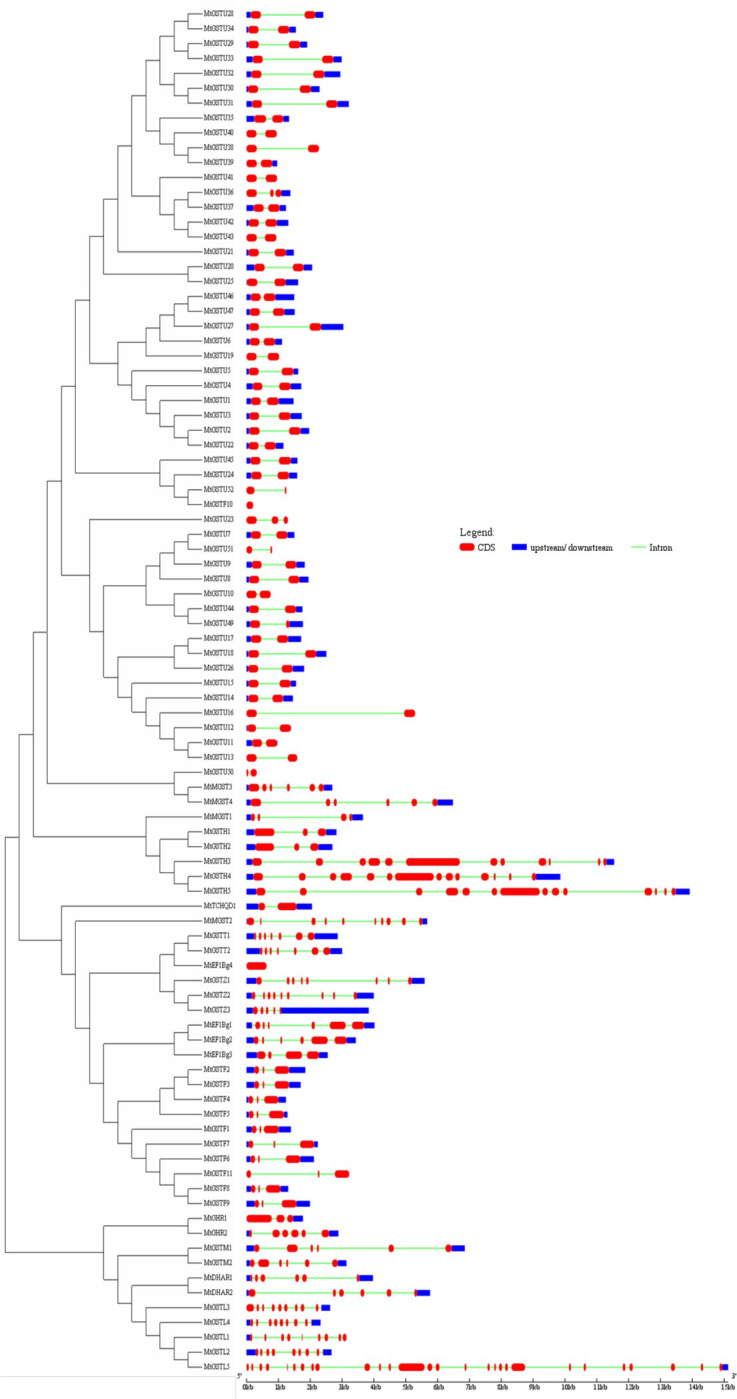
Gene structure of *MtGST* with an evolutionary relationship. The phylogenetic tree was generated using MEGA7. Exon-intron analyses of *MtGST* genes were created with GSDS tool. Lengths of introns and exons of *MtGST* genes were exhibited proportionally. Exons are shown as red boxes and introns are shown as black lines, while the untranslated region is shown as blue boxes.

The online MEME motif search program identified 10 putative conserved motifs (more than 10 amino acids long) that were found to be present in at least three classes of the MtGSTs (S2 Table in S2 File). The motif 1–8 were specific for tau class while motif 9 and 10 were specific for phi and lambda classes. To explore the expansion of GST family members in *Medicago*, an unrooted phylogenetic tree was generated (Fig 2). The phylogenetic tree showed that each class of MtGST members clustered together to form a separate clade except MtMGST1, MtEF1Bγ4, and MtMGST2 (Fig 2). This result indicated the separation of GST classes took place before the individual family member expansion. The gene structures showed that the presence of 1–3 exons in tau, phi and TCHQD members; similar to the gene structures of these classes of GST in wheat [56]. The DHAR and metaxin classes contained 6 exons while theta, GHR, zeta and EF1Bγ classes contain 3–7 exons except for *MtEF1Bγ*4 with one exon. The exon number of lambda and hemerythrin classes contained more exons than other classes varied from 3 to 25. Maximum numbers of exon found in *MtGSTL*5 (25 exons) followed by the *MtGSTH*4 and *MtGSTH*5 with 14 exons, and *MtGSTH*3 (12 exons).

### Analysis of microsatellite markers and glycosylation sites in *MtGST*s

A major focus of genetic mapping efforts is to explore molecular markers related to particular traits. A total of 101 SSR markers were distributed among 51 out of 92 *MtGST* genes. The most abundant were mononucleotide repeats (63 occurrences), followed by dinucleotide repeats (28 occurrences), trinucleotide (6 occurrences), tetranucleotide repeats (2 occurrences), and pentanucleotide (2 occurrences) (S3 Table in S2 File). Twenty-three *MtGST* genes possessed more than one SSR marker. Furthermore, glycosylation is a crucial protein secondary structure modification which plays a critical role in determining the protein 3D conformation, function and stability [57, 58]. In the present study, 48 out of 92 MtGST proteins had the presence of predicted glycosylation sites. The maximum number of 11 glycosylation sites were found in MtGSTH3 and MtGSTH4 followed by the MtGSTL5 (9 sites), MtMGST3 and MtHSTH5 with 6 sites each (S4 Table in S2 File). Prediction score greater than 0.75 indicated the maximum possibility of glycosylation. Among the total of 117 predicted sites, only 15 sites have the prediction score equal or greater than 0.75, and thus have maximum possibility glycosylation mediated secondary structure modification (Table 3).

**Table 3:** Detailed information about the maximum possibility of glycation modification among MtGST proteins.

Protein	Position	Region	Score	Protein	Position	Region	Score
MtGSTU28	42	NLSE	0.7656	MtGSTUF5	89	NDTK	0.7894
MtGSTU29	49	NLSD	0.7566	MtGSTF8	112	NLTC	0.8051
MtGSTU31	42	NLSD	0.7719	MtGSTL1	12	NSTS	0.7783
MtGSTU32	42	NLSD	0.7616	MtMGST3	244	NITD	0.7882
MtGSTU33	42	NLSD	0.7658	MtMGST4	247	NITD	0.7551
MtGSTU34	42	NLSD	0.7641	MtGSTM2	31	NFSQ	0.8052
MtGSTU35	60	NLSE	0.7802	MtGSTH1	48	NTSS	0.7626
MtGSTU38	42	NWSQ	0.7898				

### *MtGST* transcripts showed dynamics variation in various plant developmental stages and organ differentiation

**Fig 3:**
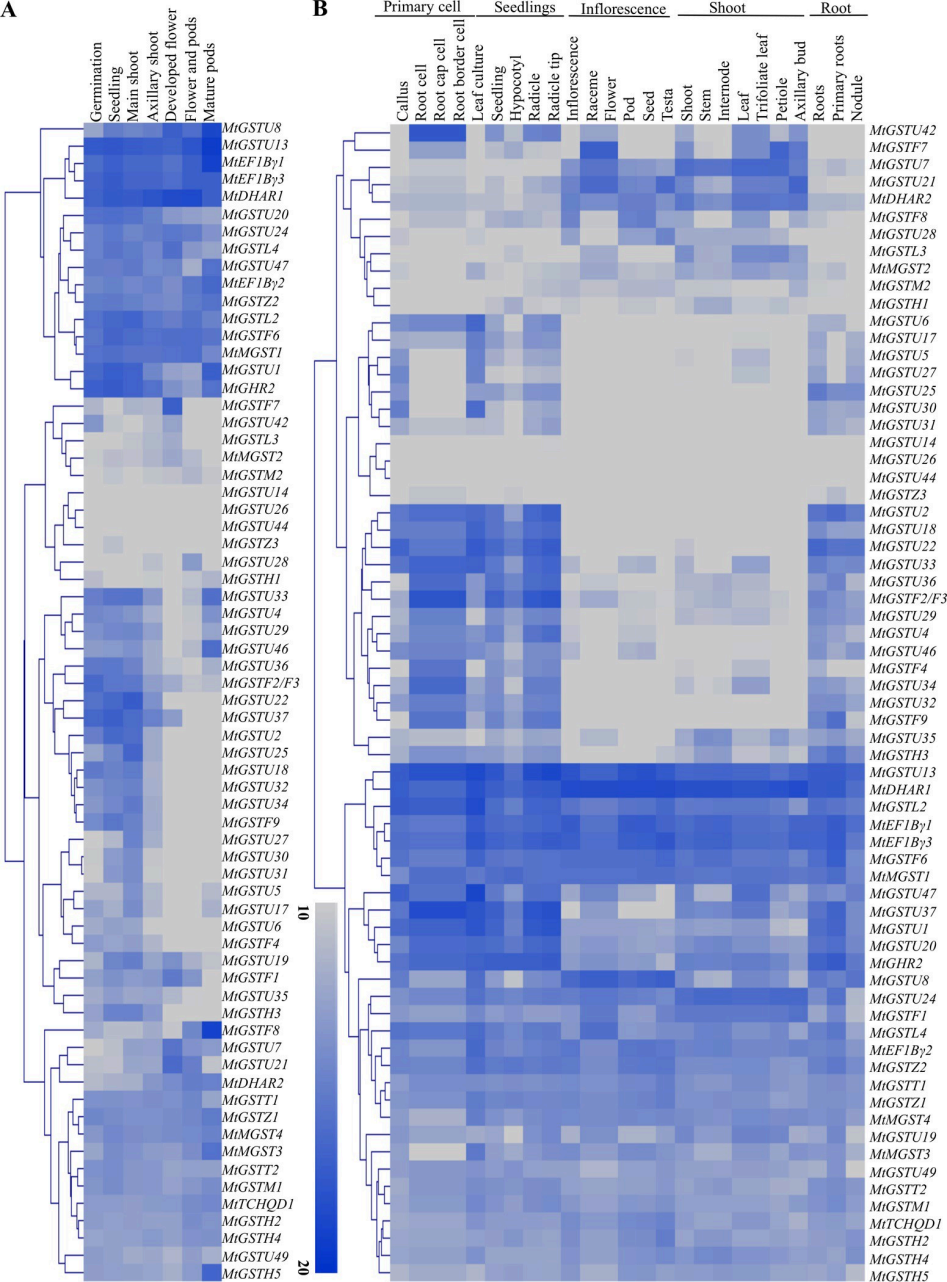
Expression profiles of sixty-eight *MtGST* transcripts in different developmental stages and anatomical tissues. (A) Expression of 68 *MtGST* genes was analyzed at five major anatomical tissues, such as primary cell, seedlings, inflorescence, shoot and roots. (B) Expression of the same 68 *MtGST* genes was analyzed at seven distinct developmental stages, such as germination, seedlings, main roots, axillary shoot, developed flower, flower and pods, and mature pods. Expression data was retrieved from genevestigator (https://genevestigator.com/gv/) and the heatmap was created with hierarchical clustering of Manhattan distance correlation using MeV software package. A colour scale is provided along with the heat map to recognize the differential pattern of expression.

To elucidate the role of *MtGST* genes, their expression was analyzed at the seven distinct developmental stages and twenty-five anatomical tissues. Expression data of 68 *MtGST* genes were analyzed and found to be expressive in all the developmental stages and anatomical tissues in a differential pattern. Based on these patterns, all these genes could be classified into three groups: i) extremely low levels of expression in almost all the tissues and organs, ii) some *MtGST*s exhibited low to medium levels of expression among different organs/tissues, and iii) some were highly expressive across all the tissues and developmental stages of its entire life cycle. Among them, *MtGSTU*13 and *MtDHAR*1 showed the maximum levels of expression in all developmental stages while *MtGSTU*8, *MtEF1B*γ1, *MtEF1B*γ3, *MtGSTU*20, *MtGSTU*24, *MtGSTL*4, *MtGSTU*47, *MtEF1B*γ2, *MtGSTZ*2, *MtGSTL*2, *MtGSTF*5, *MtMGST*1 showed high levels of expression (Fig 3A). Notably, one cluster *MtGSTU*42 to *MTGSTH*1 had extremely low levels of expression in almost all the developmental stages (Fig 3A). Similarly, one cluster of *MtGST* genes (*MtGSTU*13 to *MtGSTT*1) showed a high level of expression in almost all the analyzed tissues while another cluster *MtGSTU*8 to *MtGSTH*5 showed low to medium level of expression (Fig 3B). A cluster of four *MtGST* genes- *MtGSTU*14, *MtGSTU*26, *MtGSTU*44 and *MtGSTZ*3 possessed a very low level of expression in all the analyzed tissues. Interestingly, another cluster of *MtGST* genes- *MtGSTU*2 –*MtGSTF*9 possessed high to medium level of expression in all the analyzed tissues, except for the inflorescence and shoot specific tissues (Fig 3B). Two transcripts of *MtGSTU*13 and *MtDHAR*1 maintained a high level of expression in all the analyzed anatomical tissues similar to all the developmental stages, indicating their important roles in the plant development and tissue differentiation. Some of the *MtGST* members showed tissue-specific pattern too e.g. expression of *MtGSTU*42 is primary cell-specific while that of *MtGSTU*7 is root-specific.

### Stress-induced transcript alteration of *MtGST* genes

**Fig 4:**
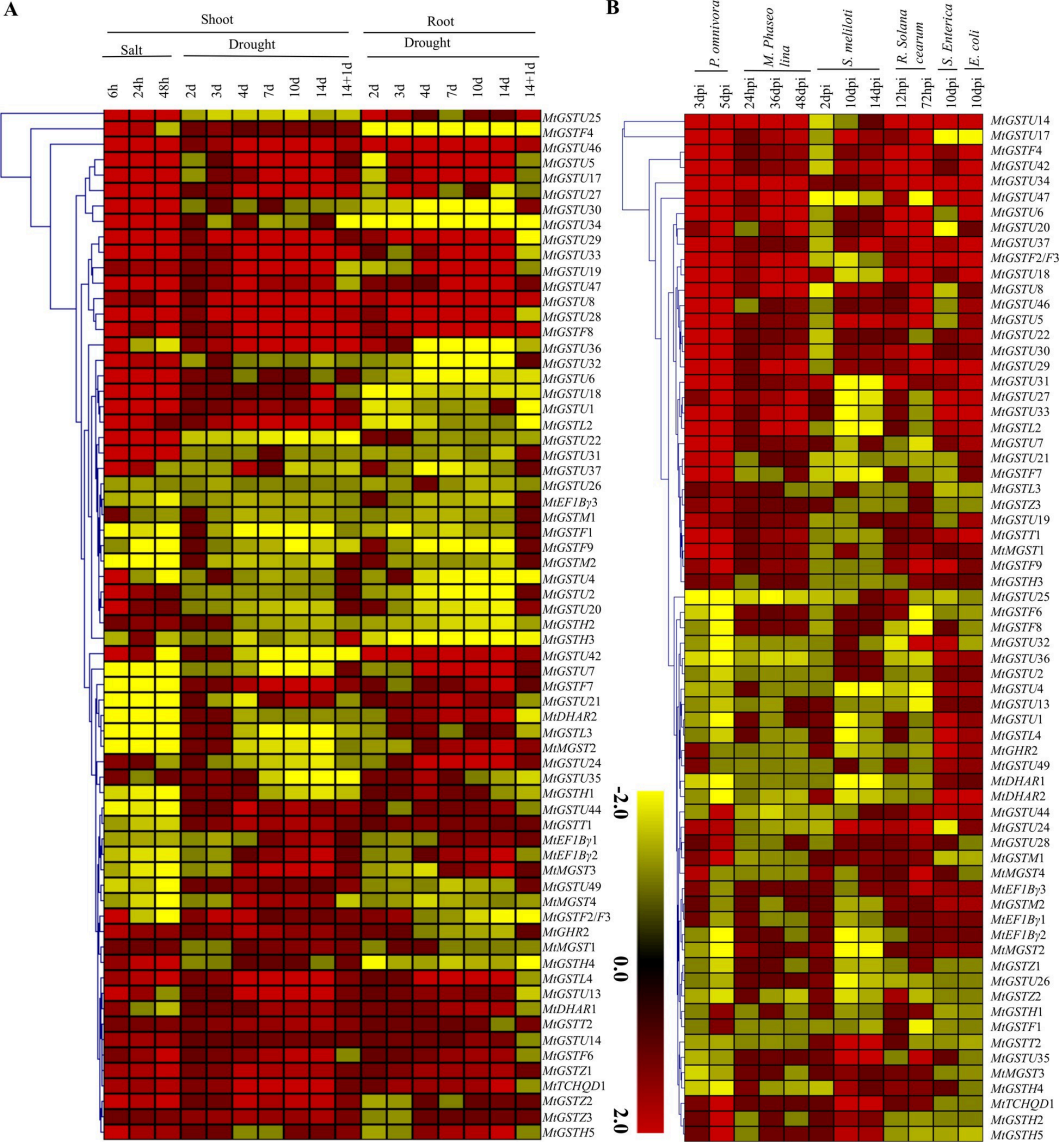
Expression pattern of *MtGST* genes under various abiotic and biotic stresses. (A) Expression pattern of sixty-eight *MtGST* genes was analyzed in response to two devesting abiotic stresses- salt and drought. Expressions were analyzed at 6h, 24h and 48h shoot samples for salinity, and samples were analyzed at 2d, 3d, 4d, 7d, 10d, 14d and 14d+1d rewatering in both shoot and root tissues for drought. (B) The expression profile was also analyzed in response to two common fungal pathogens- *Phymatotrichopsis omnivore* and *Macrophomina phaseolina*, and four infectious gram-negative bacteria—*Sinorhizobium meliloti*, *Ralstonia solanacearum*, *Salmonella enterica* and *Escherichia coli* at various day after infection (dpi). Fold change in expression as compared to control was used to generate the heatmaps with hierarchical clustering of Manhattan distance correlation in MeV software package. The colour scale provided at the bottom of the figure represents the level of expression. The stress-induced upregulation or down-regulation of *MtGST* transcripts is indicated by the red or yellow colour, respectively.

To investigate the abiotic stress-responsiveness, expression profiling of 68 *MtGST* transcripts were further analyzed in response to two different abiotic stresses viz. drought and salinity. Fold change in expression was analyzed at 6h, 24h, 48h of salt stress, while data were analyzed for 2d, 3d, 4d, 7d, 10d, 14d of drought stress and 14d drought +1d re-watering as compared to their respective 2d control expression level (S5 Table in S2 File). Most of the *MtGST* members were differentially upregulated whereas some of them were downregulated too (Fig 4A). Two clusters- *MtGSTU*46 to *MtGSTF*8 and *MtGSTL*4 to *MtGSTH*5 genes were highly up-regulated in both drought and salinity stresses in almost all the time points while one cluster *MtGSTU*37 to *MtGSTH*3 genes showed down-regulation in almost all cases with few exceptions (Fig 4A). Among them, *MtGSTU*8, *MtGSTU*17, *MtGSTF*8, *MtGSTT*2 and *MtGSTZ*1 members were highly upregulated in all cases of these two abiotic stresses. While some members showed stress-specific pattern such as *MtGSTU*44 and *MtGSTT*1 were drought specific, and *MtGSTU*2 and *MtGST*20 were salinity stress-specific (Fig 4A).

To understand the role of *MtGST* genes under biotic stresses, their expression profile was analyzed in response to two common fungal infections—*Phymatotrichopsis omnivore* and *Macrophomina phaseolina* and four gram-negative bacterial infections- *Sinorhizobium meliloti*, *Ralstonia solanacearum*, *Salmonella Enterica* and *Escherichia coli* (S6 Table in S2 File). Results suggested that one clade of *MtGST* genes- *MtGSTU*18 to *MtGSTL*2 were highly upregulated in response to all six infectious agents’ treatment except few early days after infection (dpi) time points (Fig 4B). Among the six pathogens, the maximum 43 members were upregulated in response to *E*. *coli* infection followed by 41 and 40 *MtGST* members were upregulated in response to *P*. *omnivore* and *M*. *phaseolina* infection, respectively. Although there is no specific cluster of downregulated genes, a cluster of *MtGSTU*7 to *MtGSTU*25 showed downregulation in response to almost all infections and time points. Interestingly, only *MtGSTF*4 was sharply upregulated against the infection of all pathogens at all the time points (Fig 4B).

### The putative MtGST promoters contained various cis-acting elements

**Fig 5:**
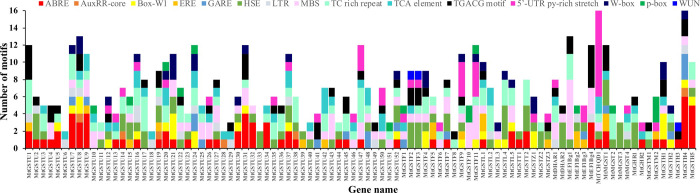
Analysis of cis-acting elements in the putative promoter of *MtGST*s. One kb 5′ upstream region of all the identified *MtGST* genes were retrieved and analyzed through the PlantCARE database to identify the presence and number of stress and hormone-responsive cis-acting regulatory elements. The number of identified motifs were plotted against a particular gene in a bar diagram. The abundance of different cis-regulatory elements on each of the promoter was represented with different colours.

To investigate the presence and position of possible cis-elements involved in the activation of stress-related genes, the putative promoter region of 68 *MtGST* genes were scanned through Plant CARE database. The analysis revealed the presence of several cis-acting elements conferring plant hormone and stress responsiveness in the promoter of *MtGST*. We have identified the presence of seven hormone-related elements such as abscisic acid-responsive (ABRE), auxin-responsive (AUXRR-core), ethylene-responsive (ERE), gibberellin-responsive (GARE and P-box), salicylic acid-responsive elements (TCA-element) and methyl jasmonate-responsive element (TGACG-motif); seven defence and stress-responsive elements such as fungal elicitor-responsive (Box-W1, W-Box), heat stress-responsive(HSE), low-temperature-responsive (LTR) elements, MYB binding site involved in drought-inducibility (MBS), stress responsiveness (TC-rich repeats), and wound-responsive element (WUN-motif) (Fig 5). The most abundant cis element found in *MtGST* promoters were stress responsiveness TC repeats (91) followed by the HSE (81), ABRE (77), and MBS (72). The promoter of *MtTCHQD*1 and *MtGSTH*5 contains the highest number of 15 cis elements followed by the promoter of *MtGSTU*8, *MtDHAR*2 and *MtEF1Bγ* with 13 members each (Fig 5 and S7 Table in S2 File). Presence of these diverse types of hormones and stress-related cis-elements in the promoter region could be directly correlated with the stress-responsive transcript alteration of *MtGST*s.

### Experimental validation of gene expression profile of ten selected *MtGSTs* genes

**Fig 6:**
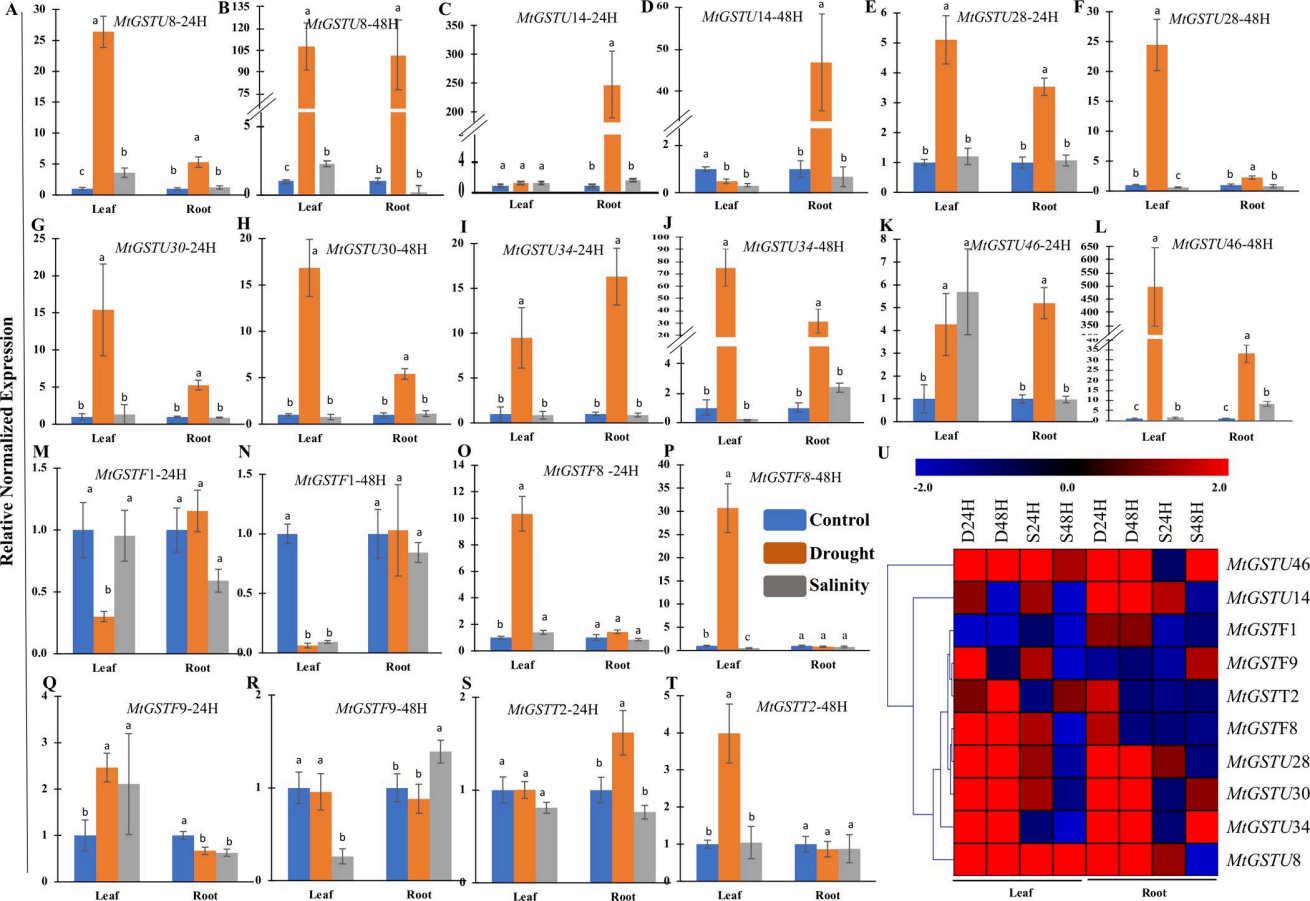
Relative normalized expression level of 10 selected *MtGST* genes in response to drought and salt stresses. Transcript abundance of ten selected *MtGST* genes were analyzed in the WT *Medicago* plants in response to two devastating abiotic stresses (salinity and drought) as compared to the respective control samples for 24h and 48h. Data represent (A-T) here the mean normalized expression value ± SD (n = 6). The Student’s t-test analysis indicated a significant alteration in their expression level as compared with the respective controls (marked with different letters). (U) Heatmap showed the fold change in expression of the selected genes in response to salinity and drought where red colour indicates upregulation and blue indicates downregulation as compared to controls.

To validate the expression pattern of *MtGST* genes in response to two abiotic stresses (drought and salinity) and minimize the variation of control, time points and tissue selection; we have analyzed the expression of ten selected stress responsive *MtGST* genes in both leaf and root tissues using the same control sample through qRT-PCR. The real-time PCR expression profile of the selected genes reveal good correlation with the microarray data. Under drought stress, most of the *MtGST* genes were highly upregulated in both shoot and root tissue samples. The expression of *MtGSTU*8, *MtGSTU*28, *MtGSTU*30, *MtGSTU*34, *MtGSTU*46 genes were upregulated both in leaf and root tissues after 24 and 48 hours of the drought treatment (Fig 6). The expression of *MtGSTU14* was highly upregulated in the root tissues on drought stress at both time points (Fig 6C and 6D), while the expression of the *MtGSTU46* gene is highly upregulated in the leaf tissues after 48 hours of drought stress (Fig 6L). This pattern of expression suggesting the root- or shoot-specific expression pattern of *MtGSTU14* and *MtGSTU46* genes to drought, respectively. The expression of MtGSTF8 showed drastic upregulation of more than 10 folds in shoot tissue at both the time points, while the expression in root is unaltered (Fig 6, O-P). Moreover, the expression of *MtGSTF1* gene downregulated in leaf tissues after both 24 and 48 hours of treatment while its expression in root tissues remained unchanged under drought stress. The *MtGSTT2* transcript showed higher expression in leaf tissues only after 48 hours of treatment (Fig 6T) and in root tissues only after 24 hours of drought stress (Fig 6S). The expression of the *MtGSTF9* gene upregulated after 24 hours for both treatments in leaf tissues but downregulated in root tissues. *MtGST*s are more expressive towards drought stress as compared with the salinity stress. Only few of the selected genes showed sharp enhancement/deregulation in response to salinity at both 24h and 48h time points. *MtGSTU*46 showed more than 5 folds upregulation in response to salinity at 24h stress treatment (Fig 6K). Similarly, the expression of *MtGSTF9* upregulated in the leaf tissues at 24h and in the root tissues after 48h of salt stress. Overall, drought treatment influences upregulation for most of the analyzed genes, while salt stress does not affect the expression of most of the targeted genes, that is clearly visible from the heatmap generated based on fold change in expression (Fig 6U).

### MtGSTU17 showed the lowest binding affinity towards GSH and CDNB

**Fig 7:**
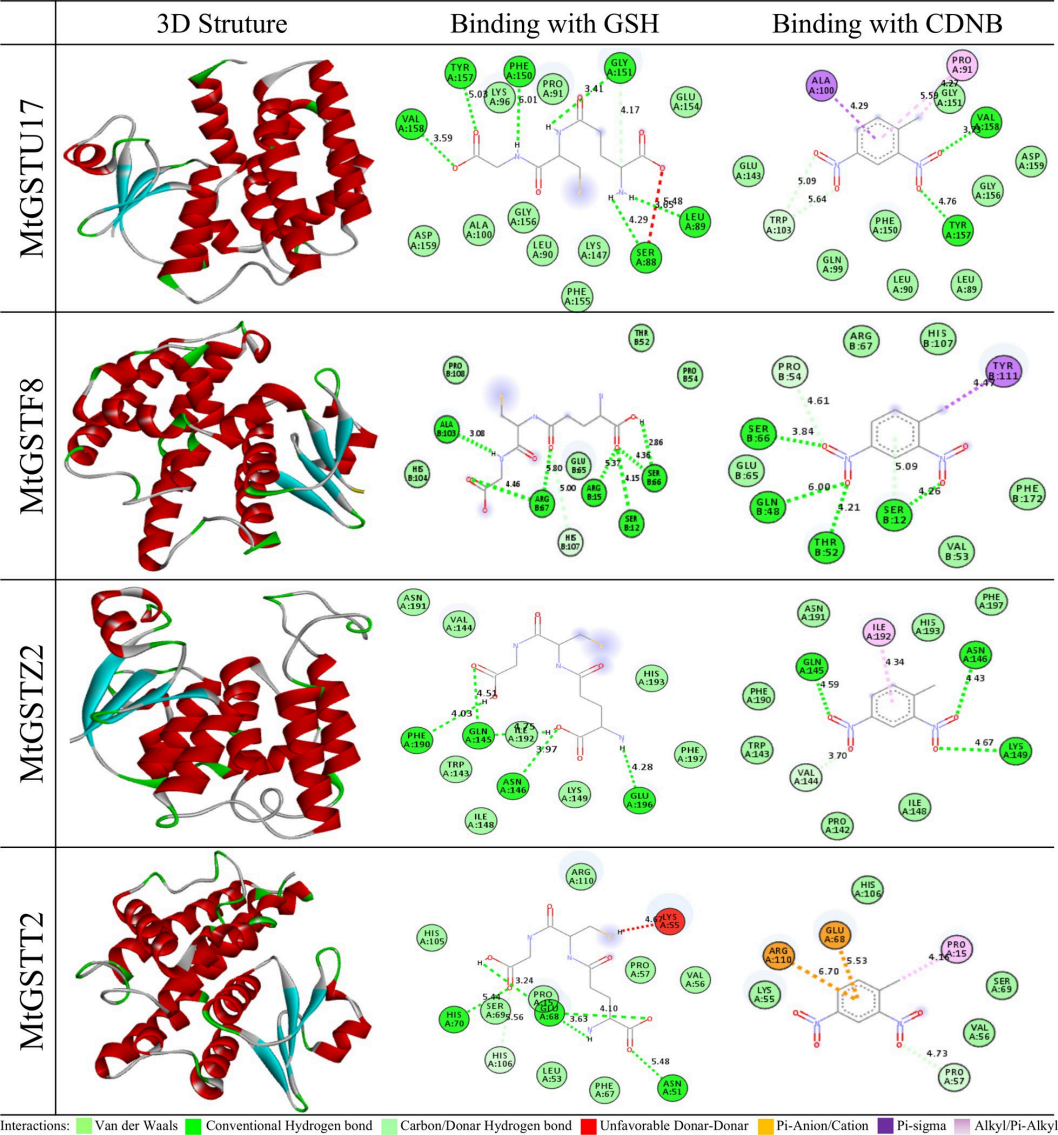
Homology modelling and molecular docking of four highly stress-responsive proteins. Four MtGST proteins (MtGSTU17, MtGSTF8, MtGSTZ2 and MtGSTT2) were used for the molecular docking with two substrate glutathione (GSH) and 1-Chloro-2,4-dinitrobenzene (CDNB). The first column represents the predicted 3D structure, the second and third column represents the 2D interaction of each protein with GSH and CDNB, respectively. The green, light green, pink, purple Orange and red spheres represent residues involved in the hydrophobic interactions, carbon-hydrogen bond interactions, Pi-alkyl interactions, Pi–cation interactions and unfavorable acceptor–acceptor interactions, respectively.

Among the highly stress-responsive *MtGST* members, ten proteins- six from tau class, one from phi class, two from zeta class and one from theta class were used in the molecular docking study to determine their affinity towards the well-known substrates of GST- GSH and CDNB (S1 Fig in S1 File). Docking scores showed that MtGSTU17 has the lowest binding energy with GSH (–5.7 kcal/mol) and CDNB (-6.5 kcal/mol) followed by MtGSTU8 with a binding energy –5.7 kcal/mol for GSH and -6.0 kcal/mol for CDNB (Table 4). Similarly, MtGSTZ2 showed a higher binding affinity with GSH (–5.2 kcal/mol) and CDNB (-5.6 kcal/mol) followed by MtGSTT2 and MtGSTF8 with GSH (-5.4 kcal/mol, - 4.8 kcal/mol) and with CDNB (-5.1 kcal/mol, - 5.2 kcal/mol), respectively in case of all classes (Table 4). The lower binding energy with its substrates of MtGSTU8 (S2 Fig in S1 File) could be directly correlated with its higher specific activities of 5.93 ±0.17 μmol/min/mg. Specific hydrogen bonds and hydrophobic interactions between four lowest binding affinity providing proteins with those ligands were analyzed using Discovery studio program. The best-scored protein MtGSTU17 stabilized with GSH within the binding pocket by forming six hydrogen bonds with Ser88, Leu89, Phe150, Gly151, Tyr157, Val158 and one hydrophobic bond with Ser88 and their bond length were 4.29, 3.65, 6.01, 3.41, 5.03, 3.59 and 5.48 A˚, respectively. Besides, MtGSTU17-CDNB docked complex formed four hydrogen bonds with Trp103, Tyr157 and Val158 while two alkyl bond and one pi-sigma bond with Pro91 and Ala100, respectively (Fig 7 and S8 Table in S2 File). Molecular dynamic (MD) simulations were performed separately for MtGSTU17-GSH and MtGSTU17-CDNB complexes to understand their structural details, conformational behavior, stability, and flexibility of the protein-ligand docked complex. In the elastic graph, each dot represents one spring between the corresponding pair of atoms, where the darker greys indicate stiffer springs and vice versa (S3 Fig in S1 File). The analyzed protein atoms involved in springs were less in both docked complex, which mainly depends on the protein atoms in amino acid residues.

**Table 4:** Predicted binding affinity (Kcal/mol) of selected MtGST proteins against reduced glutathione (GSH) and 1-chloro 2,4 dinitrobenzene (CDNB).

SL No	Protein name	Binding affinity (Kcal/mol)		Centre Grid Box (Points in X, Y, Z-axis)	Size (Points in X, Y, Z-axis)	Specific activities (μmol/ min/mg) to CDNB (Taken from Han et al., 2018 [33])
		GSH	CDNB			
1	MtGSTU8	-5.1	-6.0	-57.021 × 12.472 × 2.446	54 × 34 × 56	5.93 ±0.17
2	MtGSTU17	-5.7	-6.5	-56.413 × -14.502 × -4.882	56 × 36 × 48	0.20±0.01
3	MtGSTU28	-4.9	-5.1	17.306 × -24.944 × 5.639	50 × 54 × 46	0.08 ± 0.01
4	MtGSTU29	-4.8	-5.1	25.972 ×- 16.722 × 26.639	54 × 48 × 46	0.16 ± 0.01
5	MtGSTU46	-4.8	-5.2	23.889 × -5.167 × 18.778	54 × 50 × 46	No activity detected
6	MtGSTU47	-5.1	-5.4	24.472 × -1.972 × 18.111	54 × 56 × 48	2.71±0.06
7	MtGSTF8	-4.8	-5.2	-40.917× 59.772 ×- 2.806	52 × 46 × 52	Analysis not performed
8	MtGSTZ2	-5.2	-5.6	12.333 × 23.139 × 88.750	56 × 46 × 46	Analysis not performed
9	MtGSTZ3	-4.5	-5.8	20.639 × 25.417 × 89.833	42 × 36× 52	Analysis not performed
10	MtGSTT2	-5.4	-5.1	20.333 × 35.389 × -5.528	54 × 48 × 52	Analysis not performed

## Discussion

Adverse environmental conditions including abiotic and biotic stresses induce the production of ROS [11] which damage cellular macromolecules such as proteins, lipids, nucleic acids and cell membranes. To adapt this condition, plants have developed various physiological, chemical, and enzymatic defence mechanisms which help in their avoidance and/or tolerance of stresses [59]. In such events, antioxidant enzymes involved in the major defence mechanisms. Glutathione S-transferases (GSTs) are ubiquitous, multi-functional and antioxidants protein superfamily, that have great importance for mediating the removal of stress-inducing toxic compounds from plants. GSTs were reported from plants in the 1970s, for their potential roles in protecting maize crops from a herbicide chloro-S-triazine atrazine [60, 61].

In this report, we have identified a total of ninety-two *GST* family members, each of which members contain at least one conserved domain related to GST protein (Table 1). The number of identified *GST* genes are higher in *Medicago* as compared to 79 *GST* genes in rice [62], 55 in *Arabidopsis* [63], 84 in barley [64], 42 in maize [65], 49 in *C*. *rubella* [66], 82 in radish [67], 90 in potato [68], 90 in tomato [69], 85 in pepper [70]; but fewer than wheat with 330 GSTs [56] and soybean with 126 GSTs [71]. There is no direct correlation with the number of GST family members and their respective plant genome size (Table 5). *Medicago* possessed 1.12 and 1.16 times more GST members as compared with the similar-sized genome of radish and rice, respectively. Despite the higher genome size of *Z*. *mays*, *C*. *annuum*, *H*. *annuus*, *H*. *vulgare*; they possessed a smaller number of *GST* genes as compared with *Medicago*. The possible reason behind this observation could be the identification strategies, quality of genomic sequences and identification of new classes over time (Table 5). Data for MGST, Metaxin, Hemerythrin, GST2_N is missing for most of the previously reported plant species. However, this is one of the first reports for the presence of metaxin and hemerythrin class of GST members in *Medicago*. Another thing is clear from the GST number distribution among 20 plant species in thirteen classes (Table 5), the tau and phi classes are by far the most abundant classes in plant. The lambda, DHAR, zeta and EF1Bγ classes were next in number with a variable quantity. There is only one report for the presence of 2 GST2_N (thioredoxin-like) protein in *B*. *oleracea* [72]. Subcellular localization of 21 GST proteins from *Physcomitrella patens*, representing 10 classes were tested using C-terminal GFP fusions followed by visualization using confocal microscopy in *Nicotiana benthamiana* [73]. Sixteen proteins (Four phi- PpGSTF1, PpGSTF4, PpGSTF9, and PpGSTF10; four hemerythrin- PpGSTH1, PpGSTH2, PpGSTH3, and PpGSTH7; three EF1Bγ- PpEF1Bγ1, PpEF1Bγ2, and PpEF1Bγ4; two theta- PpGSTT1 and PpGSTT3; one TCHQD- PpTCHQD2, one Ure2p-PpUre2p1; and one zeta- PpGSTZ1) showed typical cytosolic and nuclear localizations. However, PpTCHQD3-GFP showed only cytosolic localization. Similarly, PpTCHQD5 and PpGSTL1 localized in chloroplasts; and PpDHAR1 and PpGSTI1 were shown to localize in both the cytosol and chloroplasts. Thus, the localization of GSTs has been reported in various subcellular compartments including cytosol, mitochondria, endoplasmic reticulum, nucleus and plasma membrane [74]. Subcellular localization of MtGST proteins has also been predicted to cytoplasm, chloroplast, mitochondria, nucleus, plasma membrane and extracellular space using three independent tools (Table 1).

**Table 5:** Distribution of GST family members in twenty-two different plants species.

SL no.	Plant species	Genome Size	Tau	Phi	DHAR	TCHQD	Lambda	Theta	Zeta	EF1B*γ*	GHR	MGST	Metaxin	Hemerythrin	GST2_N	Total	Reference
1	*A*. *thaliana*	135 Mb	28	14	4	1	3	3	2	0	0	0	0	0	0	55	[63]
2	*C*. *rubella*	135 Mb	25	12	3	1	2	1	3	2	0	0	0	0	0	49	[66]
3	*B*. *rapa*	284 Mb	37	22	4	1	3	2	3	3	0	0	0	0	0	75	[75]
4	*M*. *truncatula*	360 Mb	52	11	2	1	5	2	3	4	2	4	2	5	0	92	Present study
5	*O*. *sativa*	372 Mb	52	17	2	1	0	1	4	2	0	0	0	0	0	79	[62]
6	*R*. *sativus*	383 Mb	43	21	5	7	2	1	3	0	0	0	0	0	0	82	[67]
7	*P*. *trichocarpa*	480 Mb	58	9	3	1	3	2	2	3	0	0	0	0	0	58	[75]
8	*C*. *pepo*	500 Mb	18	3	0	0	1	2	3	3	2	0	0	0	0	32	[76]
9	*P*. *bretschneideri*	568 Mb	36	8	4	2	5	1	3	3	0	0	0	0	0	62	[77]
10	*B*. *oleracea*	630 Mb	28	14	4	1	3	2	2	3	5	1	0	0	2	65	[72]
11	*S*. *tuberosum*	723 Mb	66	5	3	1	5	2	2	2	2	2	0	0	0	90	[68]
12	*G*. *raimondii*	880 Mb	38	7	3	1	3	3	2	2	0	0	0	0	0	59	[78]
13	*S*. *lycopersicum*	900 Mb	57	6	6	1	7	4	2	3	2	2	0	0	0	90	[69]
14	*G*. *max*	1.1 Gb	68	14	4	3	6	6	3	4	4	5	4	5	0	126	[71]
15	*G*. *arboretum*	1.75 Gb	29	6	3	1	3	3	2	2	0	0	0	0	0	49	[78]
16	*Z*. *mays*	2.5 Gb	28	12	0	0	0	0	2	0	0	0	0	0	0	42	[65]
17	*C*. *annuum*	3.48 Gb	59	6	2	1	4	4	2	2	3	2	0	0	0	85	[70]
18	*H*. *annuus*	3.6 Gb	6	3	0	0	2	1	2	0	0	0	0	0	0	14	[68]
19	*H*. *vulgare*	4.6 Gb	50	21	2	1	2	1	5	2	0	0	0	0	0	84	[64]
20	*T*. *aestivum*	15 Gb	200	87	5	3	14	3	13	5	0	0	0	0	0	330	[56]

Species-specific gene family expansion in plants often arises as a result of tandem duplications, segmental duplications, whole-genome duplications, and interspecific hybridizations that can facilitate the evolution of functional diversity [79]. Among the functional diversities, many of the plants tend to duplicate their genes to adapt with different adverse conditions and developmental processes [80]. We have identified 7 pairs of duplicated genes in *MtGST* family, and all of them were found to be duplicated segmentally (Table 2). Most of the *MtGSTs* member exhibited similar gene structure within the same phylogenetic class, and the significant differences among the twelve classes indicated their evolutionary relationship (Fig 2). The position and numbers of intron/exon were found to be conserved in the different classes of GST (Fig 2). Usually plant GST of phi class contain three exons, tau class has two exons, zeta GSTs have ten exons, and theta group has six exons [81]. *P*. *patens* TCHQD GSTs has one to three introns, and *PpDHAR* contained four to seven introns [73]. However, the position and numbers of intron were found to be conserved in the N-terminal domain of GSTs [73]. The intensity of gene divergence could be corelated with the extensive rates of intron gain/loss in the gene structure. In the eukaryotic genome, microsatellites have widely distributed that exhibit taxon-specific variations in motif structure, genomic location, and frequency [82]. Identification of mutant SSR markers is effective in investigating the genetic variation and mapping. The identified mononucleotide repeats (62.37%), dinucleotide repeats (27.72%) and trinucleotide repeats (5.9%) could be used for the identification genotypes and presence of specific GST member.

The expression patterns of *GST* genes in different tissues had been described in many species, such as rice [62], pepper [70], and tomato [69]. Expression pattern of *MtGSTs* is found to be tissue and developmental stage specific. Similarly, four *GST* genes (*SlDHAR*2, *SlGSTF*2, *SlEF1Bγ*1, and *SlEF1Bγ*3) showed the maximum level of expression in seedlings, seed, pericarp, placenta, petal, ovary, flowers, and fruits of tomato [69]. In sunflower, six *GST* genes were mainly expressed in leaves, four in seeds, and two each in flowers and roots among the 14 identified members [68]. Expression of *GST* genes had also been found to be highly specific towards tissue and developmental stages in *Arabidopsis* [25] and rice [62]. *GST* transcripts were found to be upregulated under different abiotic and biotic stresses revealed that the function of *GST* transcript and its protein to mitigate the stress response. Among *MtGST* members, *MtGSTU*8, *MtGSTU*17, *MtGSTU*28, *MtGSTU*29, *MtGSTU*46, *MtGSTU*47, *MtGSTF*8, *MtGSTZ*2, *MtGSTZ*3 and *MtGSTT*2 were highly upregulated in two abiotic conditions. Drought treatment induces strong transcript enhancement in both root and shoot tissues within 24h as compared with salinity stress (Fig 6). Most of the abiotic stresses induce the level of reactive oxygen species and consequent generate oxidative stress. Here, salinity and drought stress treatment at *Medicago* leaves enhance the endogenous levels of H_2_O_2_ significantly as compared with the control leaves for 24h and 48h (S4 Fig in S1 File). Thus, the upregulation of *MtGST* transcripts (Fig 6) could be directly corelated with the H_2_O_2_ generation of the respective stress treatment (S4 Fig in S1 File). One of the highly drought responsive members, MtGSTU8, possessed a significantly higher specific activity (μmol/min/mg) against CNDB and 4-Chloro-7-nitrobenzofurazan (NBD-CI) as compared with other members [33].

To improve the abiotic stress resistance crops, we need a deep understanding of the structural and enzymological properties of the predicted residues involved in GSH and toxic substance binding. After analyzing, the ten maximum abiotic stress-responsive members, MtGSTU8 and MtGSTU17 were found to have the lowest binding energy with GSH and CDNB (Table 4). A study on substrate binding residues of OsGSTU17 showed that Lys42, Val56, Glu68, and Ser69 are the critical components of G-site, while Pro16, Met17, Asn109, Leu112, Tyr113, Phe116, Trp161, Phe162, and Trp165 are the critical residues of H-site [83]. Intermolecular interaction of MtGSTU17-GSH happened at the residues of Ser88, Leu89, Phe150, Gly151 Tyr157, Val158, while CDNB binds with Pro91, Ala100, Trp103, Tyr157, Val158 residues. The type and position of amino acid interacting at G-sites and H-sites were found to be conserved among OsGSTU17 and MtGSTU17. The role and importance of specific interacting residues with ligands in the GST active sites could be further analyzed using site-directed mutagenesis.

## Conclusion

To reiterate, a comparative genome-wide analysis of *GST* gene family was performed in a model legume plant, *Medicago* and identified ninety-two members with four new families. All the identified members were further investigated for their classification, transcript structure, evolution, conservation, stress responsiveness and putative function. The expression profiles of *MtGST* genes from microarray data revealed that most of the *MtGST* transcripts were highly expressed in developmental stages and anatomical tissues. Few of the selected *MtGST* transcripts were found to be extremely upregulated in response to drought stress. Finally, molecular docking study confirmed the conservance of substrates (GSH and CDNB) binding sites to GST during the detoxification reaction.

## Supporting information

S1 File [50]. (PDF)

S2 File (XLSX)
