# Supplementary material for: Genome-wide identification and expression profiling of glutathione S-transferase family under multiple abiotic and biotic stresses in Medicago truncatula L
Source: PLoS One. 2021 Feb 19;16(2):e0247170. doi: 10.1371/journal.pone.0247170 (PMC7894904; doi:10.1371/journal.pone.0247170)
Supplement: S1 File — (PDF) [file pone.0247170.s001.pdf]

Genome-wide identification and expression profiling of glutathione S-transferase family under multiple abiotic and biotic stresses in *Medicago truncatula* L.

Short title: Genome-wide identification and expression profiling of *MtGST* genes

Md Soyib Hasan[1], Vishal Singh[2], Shiful Islam[1,3], Md. Sifatul Islam[1], Raju Ahsan[1], Amita Kaundal[2], Tahmina Islam[4], Ajit Ghosh[1,*]

[1]Department of Biochemistry and Molecular Biology, Shahjalal University of Science and Technology, Sylhet-3114, Bangladesh;

[2]Department of Plants, Soils, and Climate, College of Agriculture and Applied Sciences, Utah State University, Logan Utah, USA;

[3]Present address: Department of Biological Sciences, University of Alberta, Edmonton, Alberta, Canada;

[4]Department of Botany, University of Dhaka, Dhaka-3114, Bangladesh.

*To whom correspondence should be addressed:

Ajit Ghosh Ph.D.,

E-mail: ajitghoshbd@gmail.com, aghosh-bmb@sust.edu

Supplementary Methods:

DAB staining

To measure the generation of hydrogen peroxide, a reactive oxygen species, DAB (3,3'-diaminobenzidine) staining of *Medicago* leaves under different treatment conditions was done following a method given by Daudi and O'Brien [50]. Three leaves from three individual plants in each treatment group were taken after 24 and 48 hours of treatment and placed in 2 ml of 10mM Na_2HPO_4 (control for DAB) as well as 1mg ml^{-1} DAB solution in a 12 well plate. The samples were shaken for 5 hours. After 5 hours, the solution in wells was replaced with a bleaching solution, and plates were placed in a boiling water bath for 15 minutes. The bleaching solution was replaced with a fresh bleaching solution after cooling down the plates, and pictures of the leaves were taken.

Molecular dynamics simulation study

The molecular dynamics simulation study of MtGSTU17 was performed using the online server iMODS (http://imods.chaconlab.org/) [55] with default parameters. The server provides the values of deformability, B-factor (mobility profiles), eigenvalues, variance, co-variance map, and elastic network of each enzyme-substrate complex.

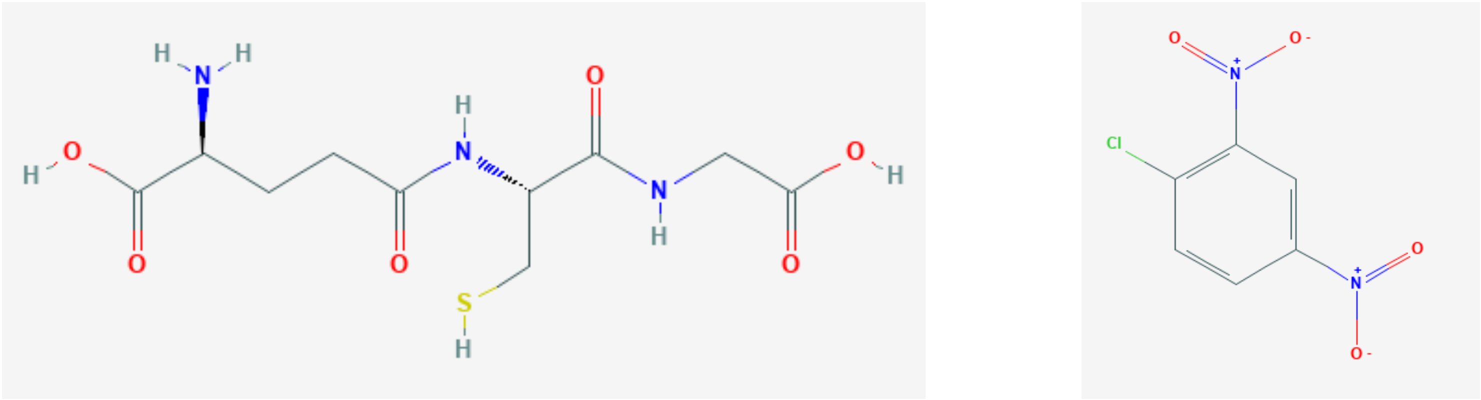

A B

Figure S1. Three dimensional structures of GSH and CNDB. Three-dimensional chemical structure of two well-known substartes of GST were retrieved from the PUBCHEM compound database (http://www.pubchem.ncbi.nlm.nih.gov) as SDF file. The molecular docking of MtGST proteins were performed against reduced glutathione (A) and 1-Chloro-2,4-dinitrobenzene (B). A. GSH (Docking Score) (PUBCHEM CID :124886) B. CDNB (PUBCHEM CID: 6).

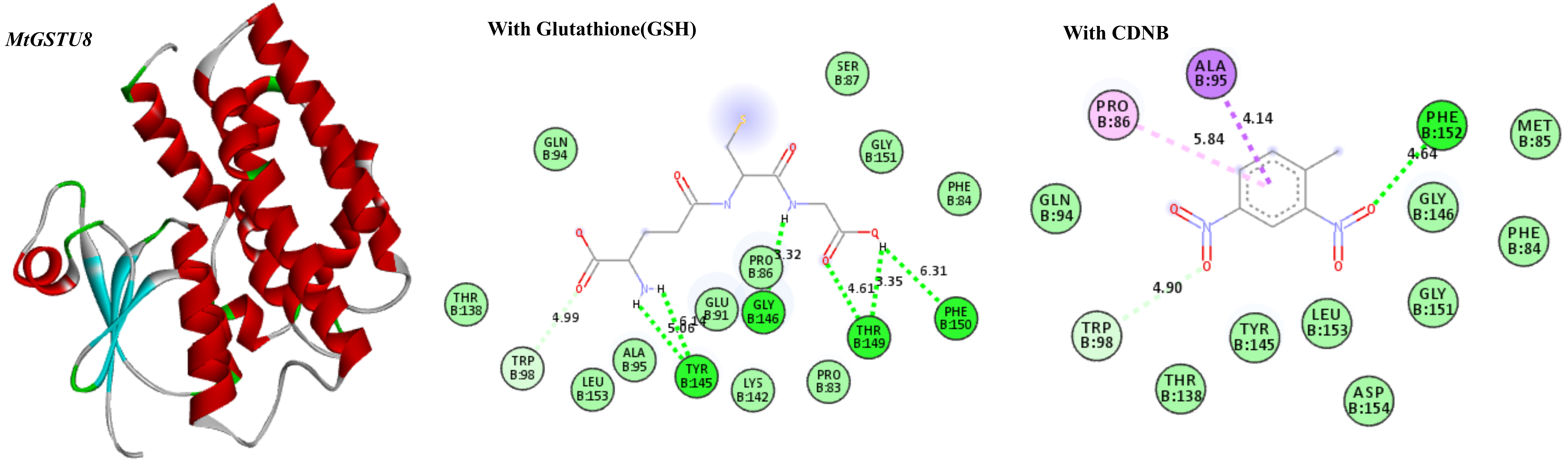

Figure S2. Homology modeling and molecular docking of MtGST8. The structure of MtGSTU8 was built using the template of Glycine max GSTU (PDB: 4TOP). The molecular docking of MtGST8 protein was performed against two substrates- reduced glutathione (GSH) and 1-Chloro-2,4-dinitrobenzene (CDNB).

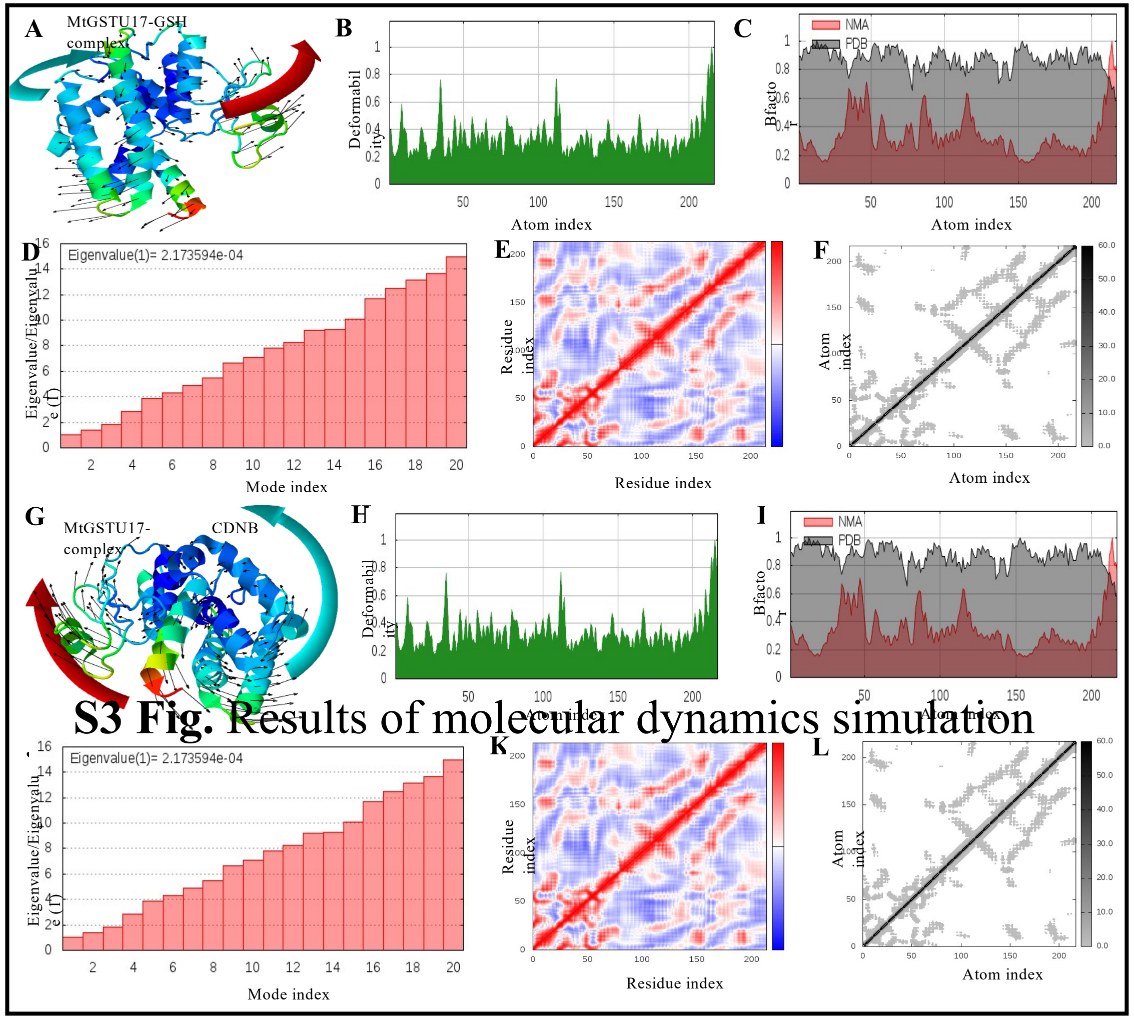

Figure S3. Results of molecular dynamics simulation. Results of MDS of MtGSTU17-GSH and MtGSTU17-CDNB docked complex. (A) NMA mobility, (B) deformability, (C) B-factor, (D) eigenvalues, (F) covariance map (correlated (red), uncorrelated (white) or anti-correlated (blue) motions) and (G) elastic network (darker gray regions indicate more stiffer regions) of MtGSTU17 – GSH complex, (H) NMA mobility, (I) deformability, (J) B-factor, (K) eigenvalues, (L) covariance map (correlated (red), uncorrelated (white) or anti-correlated (blue) motions) and (M) elastic network (darker gray regions indicate more stiffer regions) of MtGSTU17 – CDNB complex.

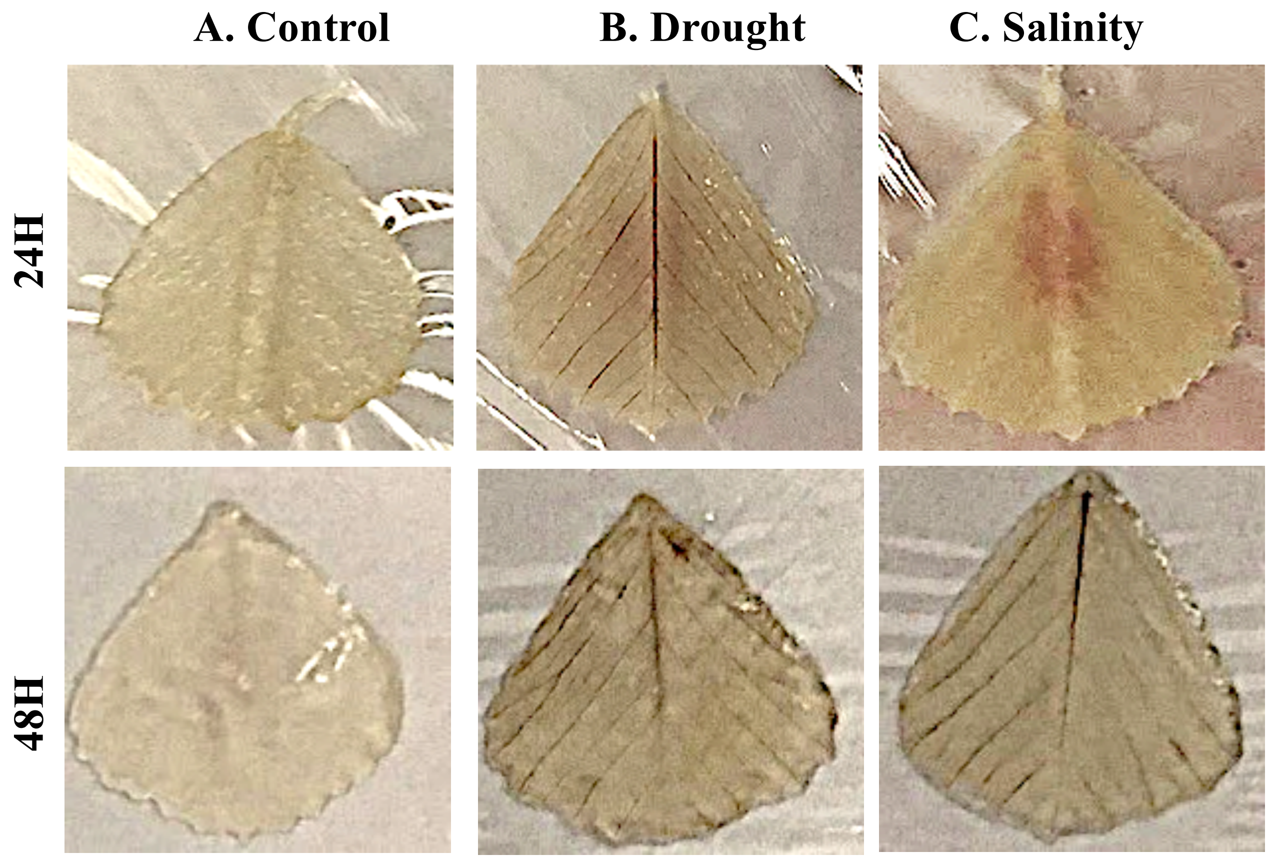

Fig S4. Detection of H_2O_2 accumulation in *Medicago* leaves under abiotic stress. *Medicago* seedling were irrigated with Peter's solution or 300mM Mannitol or 180mM NaCl to mimic control (A) or drought (B) or salinity (C) conditions, respectively. The leaf samples were collected after 24 and 48 hours of the treatment from three individual plants in each treatment group for DAB staining.
